# Associations of Y chromosomal haplogroups with cardiometabolic risk factors and subclinical vascular measures in males during childhood and adolescence

**DOI:** 10.1016/j.atherosclerosis.2018.04.027

**Published:** 2018-07

**Authors:** Linda M. O'Keeffe, Laura D. Howe, Abigail Fraser, Alun D. Hughes, Kaitlin H. Wade, Emma L. Anderson, Debbie A. Lawlor, A. Mesut Erzurumluoglu, George Davey-Smith, Santiago Rodriguez, Evie Stergiakouli

**Affiliations:** a MRC Integrative Epidemiology Unit at the University of Bristol, Oakfield House, Oakfield Grove, Bristol, BS82BN, UK; b Population Health Sciences, Bristol Medical School, Oakfield House, Oakfield Grove, Bristol, BS82BN, UK; c MRC Unit of Lifelong Health & Aging at UCL, Institute of Cardiovascular Science, University College London, London, WC1E6BT, UK; d Genetic Epidemiology Group, Department of Health Sciences, Centre for Medicine, University Road, University of Leicester, LE1 7RH, UK; e School of Oral and Dental Sciences, University of Bristol, UK

**Keywords:** Y chromosome, Childhood, Adolescence, Cardiometabolic, Vascular

## Abstract

**Background and aims:**

Males have greater cardiometabolic risk than females, though the reasons for this are poorly understood. The aim of this study was to examine the association between common Y chromosomal haplogroups and cardiometabolic risk during early life.

**Methods:**

In a British birth cohort, we examined the association of Y chromosomal haplogroups with trajectories of cardiometabolic risk factors from birth to 18 years and with carotid-femoral pulse wave velocity, carotid intima media thickness and left ventricular mass index at age 18. Haplogroups were grouped according to their phylogenetic relatedness into categories of R, I, E, J, G and all other haplogroups combined (T, Q, H, L, C, N and O). Risk factors included BMI, fat and lean mass, systolic blood pressure (SBP), diastolic blood pressure, pulse rate, triglycerides, high density lipoprotein cholesterol (HDL-c), non-HDL-c and c-reactive protein. Analyses were performed using multilevel models and linear regression, as appropriate.

**Results:**

Y chromosomal haplogroups were not associated with any cardiometabolic risk factors from birth to 18 years. For example, at age 18, the difference in SBP comparing each haplogroup with haplogroup R was −0.39 mmHg (95% Confidence Interval (CI): −0.75, 1.54) for haplogroup I, 2.56 mmHg (95% CI: −0.76, 5.89) for haplogroup E, −0.02 mmHg (95% CI: −2.87, 2.83) for haplogroup J, 1.28 mmHg (95% CI: −4.70, 2.13) for haplogroup G and −2.75 mmHg (95% CI: −6.38, 0.88) for all other haplogroups combined.

**Conclusions:**

Common Y chromosomal haplogroups are not associated with cardiometabolic risk factors during childhood and adolescence or with subclinical cardiovascular measures at age 18.

## Highlights

• Common Y chromosomal haplogroups are not associated with cardiometabolic risk factors from birth to age 18.
• Common Y chromosomal haplogroups are not associated with cardiovascular structure and function at age 18.
• Common Y chromosomal haplogroups are not associated with cardiometabolic risk in males during early life.

## 1 Introduction

Sex differences in cardiometabolic risk are well-established but are poorly understood [[1], [2], [3]]. The remaining lifetime risk of cardiovascular disease (CVD) at age 40 is two in three for males compared with one in two for females [4]. Males also develop type 2 diabetes mellitus (T2DM) at younger ages and at lower levels of adiposity than females [5,6]. Sex differences in lifestyle and hormones are likely to contribute to sex differences in cardiometabolic risk [7]. The sex chromosomes, including the male Y chromosome may also play an important role in cardiometabolic disease. However, because only 3% of Y chromosomal ancestral genes have survived genetic decay over time compared to the X chromosome and autosomes [8,9], the role of the Y chromosome in cardiometabolic disease has often been overlooked. Furthermore, the role of the Y chromosome has often been perceived as one limited to sex determination and reproductive function despite evidence that many genes on the Y chromosome are involved in other non-reproductive biological processes, and hence may be important to male health beyond reproduction [1,10,11]. In addition, due to the complexities of including the sex chromosomes in genetic association studies (including reduced power due to the use of sex-specific analyses, poor genotyping accuracy on current genome-wide arrays and quality control issues) [1], the Y chromosome is often excluded from Genome Wide Association Studies (GWAS) to simplify analyses. Thus, the association between the Y chromosome and cardiometabolic disease risk in males across the life course remains poorly understood.

Recently, the association of the Y chromosome with cardiometabolic disease risk in males has been studied using Y chromosomal haplogroups, which are stable lineages of the Y chromosome derived by genotyping and mapping to branches of the Y chromosome phylogenetic tree [12]. Three studies found no evidence that common Y chromosomal haplogroups were associated with cardiometabolic risk factors or vascular outcomes in European men [[13], [14], [15]] while others have found some evidence of blood pressure differences between haplogroups [16] and a 50% increased risk of coronary artery disease (CAD) in carriers of haplogroup I compared with haplogroup R [17]. However, the association of common Y chromosomal haplogroups with cardiometabolic risk factors in early life (which are known to track into adulthood and are associated with later cardiovascular risk) has not been examined [[18], [19], [20]]. Given that the underlying pathophysiological process of atherosclerosis is already beginning during childhood [21,22], examining the association of common Y chromosomal haplogroups with cardiometabolic risk factors in early life, may provide important aetiological insights into the role of the Y chromosome in cardiometabolic risk, before pharmacological treatment of risk factors can bias associations. In addition, examining associations with risk factor change over time, may provide insights into the mechanisms underlying the role of the Y chromosome in cardiometabolic risk, if associations emerge during critical periods of growth and development in early life (such as puberty).

The objectives of this study were to examine the association between common Y chromosomal haplogroups and cardiometabolic risk factors during childhood and adolescence. To do this, we examined the associations of common Y chromosomal haplogroups (haplogroup R, haplogroup I, haplogroup E, haplogroup J, haplogroup G, and all other haplogroups combined (haplogroups T, Q, H, L, C, N and O)) with trajectories of cardiometabolic risk factors from birth to 18 years in a contemporary prospective birth cohort study in the South West of England. These included BMI (from 1 to 18 years); fat and lean mass (from 9 to 18 years); systolic blood pressure (SBP), diastolic blood pressure (DBP), pulse rate and glucose (from 7 to 18 years); triglycerides, high density lipoprotein cholesterol (HDL-c) and non-HDL-c (from birth to 18 years); c-reactive protein (CRP) (from 9 to 18 years) and measures of sub-clinical cardiovascular disease (carotid-femoral pulse wave velocity (PWV), left ventricular mass index (LVMI) and carotid intima media thickness (cIMT)) at age 18 years.

## 2 Materials and methods

### 2.1 Study participants

The Avon Longitudinal Study of Parents and Children (ALSPAC) is a prospective birth cohort study in the South West England. [23,24] Pregnant women resident in one of the three Bristol-based health districts with an expected delivery date between April 1, 1991 and December 31, 1992 were invited to participate. The study has been described elsewhere in detail. [23,24] ALSPAC initially enrolled a cohort of 14,451 pregnancies, from which 13,867 live births occurred in 13,761 women. Follow-up has included parent and child completed questionnaires, links to routine data and clinic attendance. Research clinics were held when the participants were approximately 7, 9, 10, 11, 13, 15 and 18 years old. Ethical approval for the study was obtained from the ALSPAC Ethics and Law Committee and the Local Research Ethics Committees. The study website contains details of all the data that is available through a fully searchable data dictionary http://www.bristol.ac.uk/alspac/researchers/access/ [25].

### 2.2 Data

#### 2.2.1 Genotyping of the Y chromosome

Blood samples for DNA extraction were obtained from several sources, including cord blood, whole blood, and mouthwash samples from children who did not wish to give blood. A standard phenol-cholorform extraction method was used for DNA extraction for all samples from long-term storage and a salting-out method was used for samples stored at −20 °C for less than one month. All batches of DNA extractions include a control sample to monitor recovery [26]. A total of 9,912 of the ALSPAC offspring participants were genotyped using the Illumina HumanHap550 quad genome-wide single nucleotide polymorphism (SNP) genotyping platform by Sample Logistics and Genotyping Facilities at the Wellcome Trust Sanger Institute and LabCorp (Laboratory Corporation of America) using support from 23andMe. PLINK software (v1.07) was used to carry out quality control (QC) measures [27]. For each individual, the resulting Y chromosomal genotypes (816 SNPs) were then piped into the Y-Fitter (v0.2) software, which maps genotype data to the Y chromosome phylogenetic tree built by Karafet *et al* (available online at sourceforge.net/projects/yfitter) and their respective Y chromosomal haplogroup was determined [28]. After removal of individuals with ‘false’ haplogroup determinations (i.e., individuals who did not have enough SNPs to reliably determine haplogroup), 5,080 individuals were available. SNPs with a minor allele frequency (MAF) of <1% and call rate of <95% were removed. The pseudo-autosomal SNPs (coded as chromosome 25) were removed from the analysis using the PLINK software [27]. Individuals were excluded from analysis based on having incorrect gender assignments, minimal or excessive heterozygosity (<0.320 and >0.345 for the Sanger data and <0.310 and >0.330 for the LabCorp data), disproportionate levels of individual missingness (>3%), evidence of cryptic relatedness (>10% IBD) and being of non-European ancestry (as detected by a multidimensional scaling analysis seeded with HapMap 2 individuals). EIGENSTRAT analysis revealed no additional obvious population stratification and genome-wide analyses with other phenotypes indicated a low lambda.

Individuals belonging to haplogroup R (predominantly R1b and R1a) were clustered into a single group and used as the reference group in this analysis. Individuals belonging to haplogroup I (predominantly I1 and I2), E (predominantly E1b), J (predominantly J2) and G (predominantly G2) were also clustered together, each into their own separate groups (I, E, J, and G). The remaining haplogroups were then grouped together due to their low prevalence in our cohort, listed in order of frequency: (T, Q, H, L, C, N and O). Supplementary Table 1 shows a detailed breakdown of the specific haplogroups contributing to these categories for CRP (risk factor with fewest individuals and repeated measures) and BMI (risk factor with greatest number of individuals and repeated measures).

#### 2.2.2 Anthropometry

Length (before the age of 2 years), height (from the age of 2 years) and weight data from the age of 1 year for the participants were obtained from several sources including health visitor records, questionnaires and clinics from birth to 18 years [29]. BMI was calculated as weight (kg) divided by height squared (m^2^). Whole body less head, and central fat and lean mass were derived from whole body dual energy X-ray absorptiometry (DXA) scans assessed 5 times at ages 9, 11, 13, 15, and 18 using a Lunar prodigy narrow fan beam densitometer.

#### 2.2.3 SBP, DBP and pulse rate

At each clinic (ages 7, 9, 10, 11, 12, 15 and 18), SBP, DBP and pulse rate were measured at least twice each with the child sitting and at rest with the arm supported, using a cuff size appropriate for the child's upper arm circumference and a validated blood pressure monitor. The mean of the two final measures was used.

#### 2.2.4 Blood based biomarkers of cardiometabolic risk

Non-fasting glucose was also measured at age 7 as part of metabolomics assays, using Nuclear Magnetic Resonance (NMR) Spectroscopy. In a random 10% of the cohort at age ∼9 years, fasting glucose was also available. Fasting glucose was available from clinics held when participants were 15 and 18 years old. Triglycerides, HDL-c and total cholesterol were measured in cord blood at birth and from venous blood subsequently. Samples were non-fasted at 7 and 9, with fasting measures available from clinics at 15 and 18 years. Non-HDL-c was calculated by subtracting HDL-c from total cholesterol at each measurement occasion. Trajectories of glucose, triglycerides, HDL-c and non-HDL-c were derived from a combination of measures from cord blood, fasting bloods, non-fasting bloods and NMR spectroscopy.

#### 2.2.5 Cardiovascular structure and function

Common carotid artery B-mode ultrasound images were acquired with the head rotated to 45° from the midpoint using a Zonare Z.OneUltra system equipped with a L10-5 linear transducer (Zonare Medical Systems, CA, US). Images were recorded in Digital Imaging and Communications in Medicine (DICOM) format as 10 s cine-loop files for offline analysis using the Carotid Analyser (Medical Imaging Applications, Coralville, IA). Left and right cIMT were taken to be the average of 3 end-diastolic measurements of the far-wall of the common carotid artery over a 5–10 mm length, 10 mm proximal to the bifurcation. The mean of left and right cIMT was calculated and used in analyses.

A sub-sample of study participants from the 18-year clinic underwent echocardiography using a HDI 5000 ultrasound machine (Phillips) and P4-2 Phased Array ultrasound transducer using a standard examination protocol. Left ventricular mass (LVM) was estimated according to American Society of Echocardiography (ASE) guidelines [30]. LVM measured at each age was indexed to height^2.7^ (LVMI).

Aortic stiffness (PWV) was assessed using a Vicorder device (Skidmore Medical, UK). Participants rested supine on a couch with their head raised to 30°. Real-time pulse-wave forms were recorded simultaneously from proximal (right carotid) and distal (the upper right thigh) sensor cuffs and the time delay measured. Transit distance was measured from suprasternal notch directly to the top of the thigh cuff. Measurements were taken until pressure waveforms over the carotid and thigh area were of high quality and reproducible. Three PWV measurements, within ≤0.5 m/s of each other, were averaged.

**Fig. 1:**
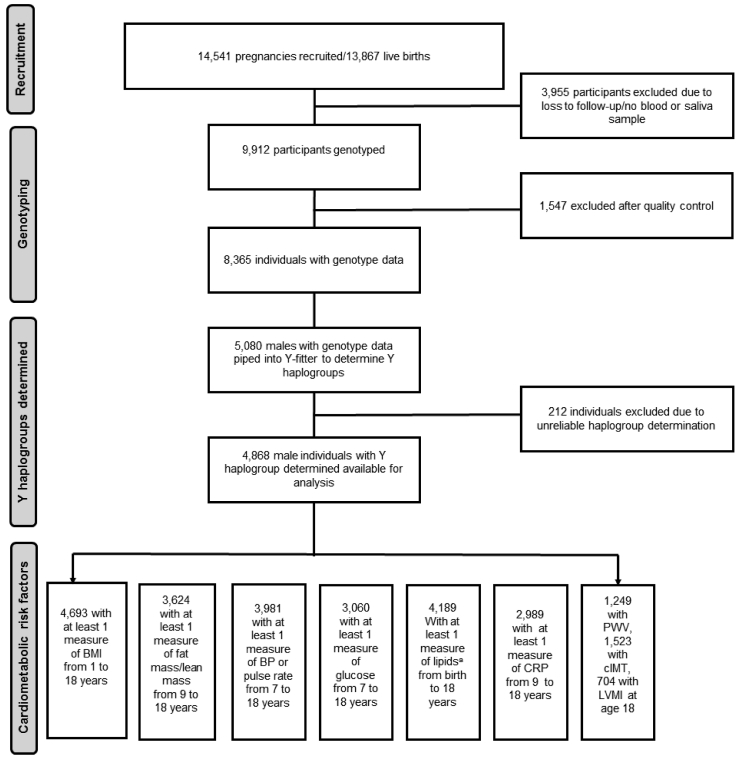
Flow diagram of study. BMI, body mass index; BP, blood pressure; cIMT, carotid intima media thickness; CRP, c-reactive protein; LVMI, left ventricular mass index; PWV, pulse wave velocity. ^a^ Lipids include triglyceride, high density lipoprotein cholesterol (HDL-c) and non-HDL-c.

Fig. 1 shows a flow diagram for the study and further details of measurement of risk factors are available in Appendix 1 of the Supplementary Material.

### 2.3 Statistical analysis

#### 2.3.1 Main analysis

We used multilevel models to examine the association between common Y chromosomal haplogroups and change in each risk factor across childhood and into adolescence [31,32]. Associations between haplogroups and risk factors measured at one time point only (PWV, cIMT, LVMI) were examined using linear regression. Multilevel models estimate mean trajectories of the risk factor while accounting for the non-independence (i.e. clustering) of repeated measurements within individuals, change in scale and variance of measures over time, and differences in the number and timing of measurements between individuals (using all available data from all eligible participants under a missing at-random assumption (MAR)) [33,34]. Linear splines, fractional polynomials and linear age terms were used in the modelling of trajectories. Linear splines allow knot points to be fitted at different ages to derive periods of change that are approximately linear. Fractional polynomials involve raising age to many combinations of powers, resulting in a wide range of possible curves and offering more flexibility than standard polynomial approaches.

We included all participants with at least one measure of the risk factor in each multilevel model, under a MAR assumption, to minimise selection bias. All trajectories except BMI and CRP (fat mass, lean mass, SBP, DBP, pulse rate, glucose, triglycerides, HDL-c and non-HDL-c) were estimated using linear spline multilevel models (with two levels: measurement occasion and individual). The optimal linear spline model for each cardiometabolic risk factor was selected based on previous work [33,35,36] or by comparing model fit statistics (Akaike's Information Criterion [37]) for several models with different knot points (placed at whole years closest to mean age at clinics due to a greater density of measures). Trajectories of BMI were modelled using fractional polynomials [38] with two levels (measurement occasion and individual), since change in BMI during childhood follows a complex pattern that cannot be appropriately modelled using linear splines. CRP was modelled using a linear age term with two levels (measurement occasion and individual). Model fit statistics for each risk factor trajectory are shown in Supplementary Tables 4 to 12. An interaction between haplogroups and each spline (in the case of linear spline multilevel models) or the fractional polynomial age terms or linear age terms (in the case of BMI or CRP) were included in the models to estimate the difference in intercepts and slopes of each cardiometabolic risk factor between the haplogroups. To allow BMI and CRP differences between haplogroups over time to be more easily interpreted, coefficients from the models were used to predict mean BMI or CRP at different ages. All trajectories were modelled in MLwiN version 2.36 [39], called from Stata version 14 [40] using the runmlwin command. [41].

In all models, age (in years) was centred at the first available measure. Values of cardiometabolic risk factors that had a skewed distribution (BMI, fat mass, triglycerides and CRP) were (natural) log transformed prior to analyses. Graphs displayed for these risk factors are in original units and derived by back transforming from the natural log scale. Differences and confidence intervals were calculated on the log-scale, which were also back-transformed and are therefore interpreted as the ratio of geometric means. We performed power calculations for risk factors with the smallest sample sizes for both i) the group with the smallest number of participants (haplogroup G) and the group of greatest clinical interest in the analysis (Haplogroup I due to its previous association with a 50% increased risk of CAD [17]). Further details of model selection and power calculations are provided in Appendix 2 and Supplementary Tables 2 and 3. Model fit statistics for each risk factor trajectory are shown in Supplementary Tables 4-12.

#### 2.3.2 Sensitivity analyses

We performed sensitivity analyses excluding all observations of lipids and glucose, for participants who reported eating in the 4 hours preceding the 15 and 18-year clinics to examine if our results were altered by the inclusion of some non-fasted bloods at these clinics. We performed sensitivity analysis restricting BMI to participants with greater than 6 measures, to examine if results were driven by participants with a greater number of measures.

## 3 Results

Table 1 shows the number of participants with available measures of cardiometabolic risk factors at each age along with the median and interquartile range (IQR) of available measures. Sample sizes for repeatedly measured risk factors ranged from 2,989 participants (5,341 repeated measures) for CRP up to 4,693 participants (45,316 repeated measures) for BMI. Sample sizes for measures of vascular structure and function measured at age 18 ranged from 704 to 1,523. Table 2 shows the frequency of each haplogroup analysed in the study. Maternal marital status, household occupational social class, maternal and paternal education and maternal smoking during pregnancy did not differ by offspring haplogroup (Supplementary Table 13).

**Table 1:** Number of participants with cardiometabolic measures at each time point.

	Birth	Age 1	Age 7	Age 9	Age 10	Age 11	Age 12	Age 13	Age 15	Age 18	Total participants	Total measures	Range of measures	Median measures (IQR)
BMI^a^			x	x	x	x	x	x	x	x	4693	45,316	1–36	12 [[9], [10], [11], [12], [13], [14], [15], [16]]
Fat/lean mass				3090		2942		2567	2153	1802	3624	12,554	1–5	4 [[3], [4], [5]]
SBP/DBP/pulse rate			3417	3230	3012	2954	2800		2179	1740	3981	19,332	1–7	6 [[5], [6], [7]]
Glucose			2138	421					1555	1372	3060	5586	1–4	2 [[1], [2], [3]]
Lipid^b^	1707		2564	2417					1575	1399	4186	9662	1–4	2 [[1], [2], [3]]
CRP				2395					1561	1385	2989	5341	1–3	2 [[1], [2], [3]]
PWV	–	–	–	–	–	–	–	–	–	1249	1249	1249	1	1
cIMT	–	–	–	–	–	–	–	–	–	1523	1523	1523	1	1
LVMI	–	–	–	–	–	–	–	–	–	704	704	704	1	1

cIMT, carotid intima media thickness; CRP, c-reactive protein; DBP, diastolic blood pressure; IQR, interquartile range; LVMI, left ventricular mass index; PWV, pulse wave velocity; SBP, systolic blood pressure.

a Measures available at each of these approximate ages and at several ages in between, but exact timing and number of BMI measures not shown as measures were available from questionnaires, routine child health records and research clinics at different mean ages from 1 to 18 years.

b Lipids include triglyceride, high density lipoprotein cholesterol (HDL-c) and non-HDL-c.

**Table 2:** Frequencies of haplogroups among participants with at least 1 measure of each risk factor.

	BMI	Fat mass/Lean mass	Blood pressure/pulse rate	Glucose	Lipids	CRP	PWV	cIMT	LVMI
Total	4667	3624	3981	3060	4186	2989	1247	1523	704
*R*^a^	3, 384 (72.0)	2627 (72.5)	2870(72.1)	2206 (72.1)	3017 (72.1)	2165 (72.4)	924 (74.0)	1127 (74.00)	523 (74.3)
*I*^a^	887 (18.9)	670 (18.5)	742 (18.6)	564 (18.4)	786 (18.8)	550 (18.4)	226 (18.1)	280 (18.4)	120 (17.0)
*E*^a^	140 (3.0)	106 (2.9)	121 (3.0)	93 (3.0)	124 (3.0)	90 (3.0)	36 (2.9)	43 (2.8)	22 (3.1)
*J*^a^	125 (2.7)	96 (2.7)	112 (2.8)	82 (2.7)	116 (2.8)	58 (1.9)	26 (2.1)	32 (2.1)	16 (2.3)
*G*^a^	86 (1.8)	68 (1.9)	74 (1.9)	61 (2.0)	79 (1.9)	81 (2.7)	24 (1.9)	26 (1.7)	17 (2.4)
*Other*^b^	45 (1.5)	57 (1.6)	62 (1.6)	54 (1.8)	64 (1.5)	45 (1.5)	13 (1.0)	15 (1.0)	6 (0.9)

cIMT, carotid intima media thickness; CRP, c-reactive protein; DBP, diastolic blood pressure; IQR, interquartile range; LVMI, left ventricular mass index; PWV, pulse wave velocity; SBP, systolic blood pressure.

a For details of the specific subgroups contributing to these larger groups see Supplementary Table 1. Haplogroup R contains predominantly R1b and R1a, I contains predominantly I2 and I1, E contains predominantly E1b, J contains predominantly J2 and G contains predominantly G2.

b The “other” haplogroup includes (listed in order of frequency): T, Q, H, L, C, N and O. Frequencies of these are also displayed in Supplementary Table 1.

**Fig. 2:**
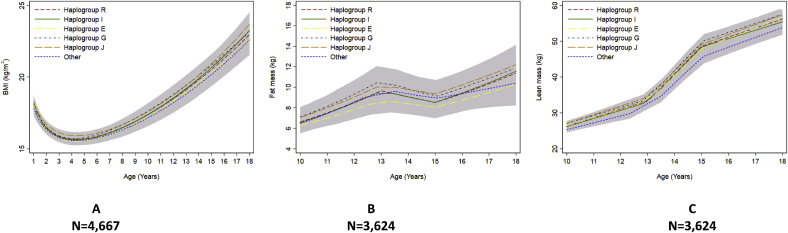
Mean predicted trajectories of anthropometry. Mean predicted trajectories of (A) BMI (1–18 years), (B) height-adjusted fat mass and (C) height-adjusted lean mass (9–18 years) by haplogroup. For details of the specific subgroups contributing to these larger groups see Supplementary Table 1. The “other” haplogroup includes (listed in order of frequency): T, Q, H, L, C, N and O. Frequencies of these are also displayed in Supplementary Table 1. Confidence intervals for all haplogroups are displayed in grey but are entirely over-lapping as the difference between the trajectories of the haplogroups spans the null value across the entire age range. Detailed results with confidence intervals are provided in Supplementary Tables 14–16.

**Fig. 3:**
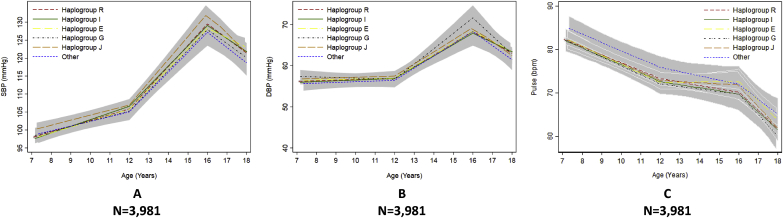
Mean predicted trajectories of blood pressure and pulse rate by haplogroup. Mean predicted trajectories of (A) SBP, (B) DBP and (C) pulse rate from 7 to 18 years by haplogroup. DBP, diastolic blood pressure; SBP, systolic blood pressure. For details of the specific subgroups contributing to these larger groups see Supplementary Table 1. The “other” haplogroup includes (listed in order of frequency): T, Q, H, L, C, N and O. Frequencies of these are also displayed in Supplementary Table 1. Confidence intervals for all haplogroups are displayed in grey but are entirely over-lapping as the difference between the trajectories of the haplogroups spans the null value across the entire age range. Detailed results with confidence intervals are provided in Supplementary Table 17.

**Fig. 4:**
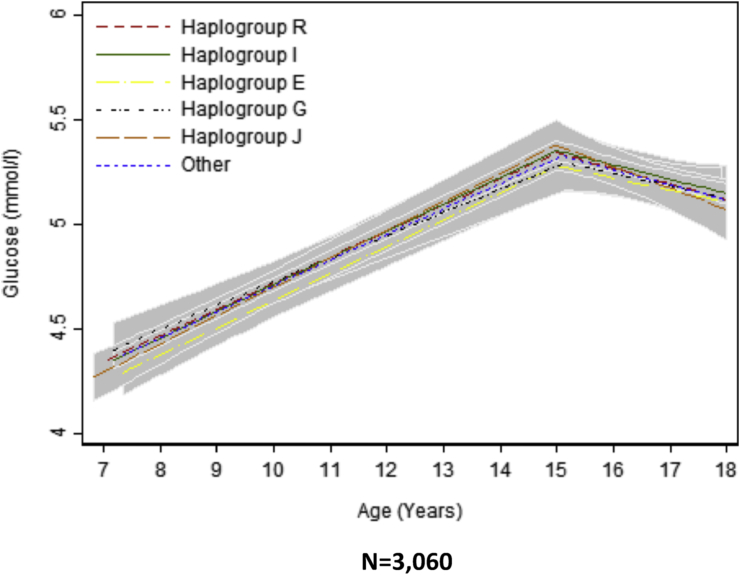
Mean predicted trajectories of glucose (7–18 years) by haplogroup. For details of the specific subgroups contributing to these larger groups see Supplementary Table 1. The “other” haplogroup includes (listed in order of frequency): T, Q, H, L, C, N and O. Frequencies of these are also displayed in Supplementary Table 1. Confidence intervals for all haplogroups are displayed in grey but are entirely over-lapping as the difference between the trajectories of the haplogroups spans the null value across the entire age range. Detailed results with confidence intervals are provided in Supplementary Table 18.

**Fig. 5:**
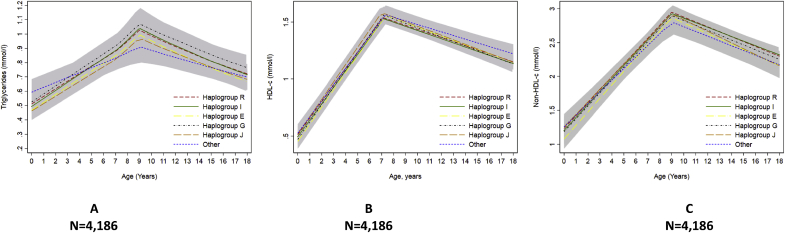
Mean predicted trajectories of lipids by haplogroup. Mean predicted trajectories of (A) triglyceride, (B) HDL-c and (C) non-HDL-c from birth to 18 years by haplogroup. HDL-c, high density lipoprotein cholesterol; non-HDL-c, non-high density lipoprotein cholesterol. For details of the specific subgroups contributing to these larger groups see Supplementary Table 1. The “other” haplogroup includes (listed in order of frequency): T, Q, H, L, C, N and O. Frequencies of these are also displayed in Supplementary Table 1. Confidence intervals for all haplogroups are displayed in grey but are entirely over-lapping as the difference between the trajectories of the haplogroups spans the null value across the entire age range. Detailed results with confidence intervals are provided in Supplementary Tables 19 and 20.

**Fig. 6:**
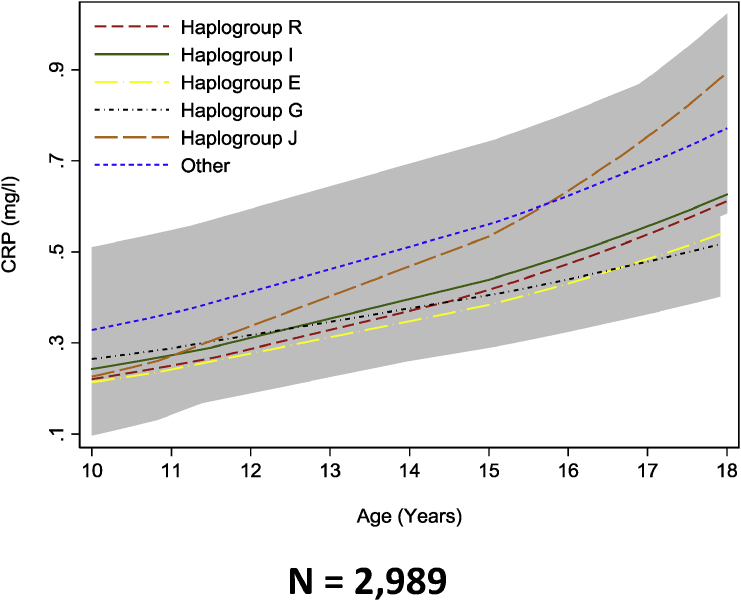
Mean predicted trajectories of CRP from 9 to 18 years by haplogroup. CRP, c -reactive protein. For details of the specific subgroups contributing to these larger groups see Supplementary Table 1. The “other” haplogroup includes (listed in order of frequency): T, Q, H, L, C, N and O. Frequencies of these are also displayed in Supplementary Table 1. Confidence intervals for all haplogroups are displayed in grey but are entirely over-lapping as the difference between the trajectories of the haplogroups spans the null value across the entire age range. Detailed results with confidence intervals are provided in Supplementary Table 21.

We found no strong evidence of a difference in the trajectories of any of the cardiometabolic risk factors across childhood and adolescence between haplogroups R, I, E, J, G and other haplogroups combined (Fig. 2, Fig. 3, Fig. 4, Fig. 5, Fig. 6 and Supplementary Tables 14–21). We also found no strong evidence that haplogroups were associated with PWV, cIMT or LVMI at age 18 (Table 3). For example, compared with haplogroup R, haplogroup J had similar log BMI at age 1 year (difference: −2.76% 95% Confidence Interval CI: −12.96%, 7.45%) and the groups remained similar at age 18 years (difference: 2.72% (95% CI: −0.72%, 5.15%), as indicated by confidence intervals that span the null value (Supplementary Table 14).

**Table 3:** Cardiovascular structure and function at age 18 by haplogroup.

Risk factor	N	Predicted risk factor at age 18	Estimate (95% CI)
Log carotid-femoral PWV (m/s)	1249		
R^a^		1.79 (1.78, 1.80)	reference
I^a^		1.79 (1.78, 1.80)	−0.00005 (−0.02, 0.02)
E^a^		1.78 (1.74, 1.81)	−0.01 (−0.05, 0.02)
J^a^		1.79 (1.75, 1.84)	0.005 (−0.04, 0.05)
G^a^		1.79 (1.75, 1.84)	0.004 (−0.04, 0.05)
Other^b^		1.77 (1.71, 1.83)	−0.02 (−0.08, 0.04)
Mean cIMT (mm)	1523		
R^a^		0.48 (0.48, 0.49)	reference
I^a^		0.48 (0.48, 0.49)	−0.002 (−0.01, 0.003)
E^a^		0.48 (0.47, 0.49)	−0.005 (−0.019, 0.009)
J^a^		0.49 (0.47, 0.50)	0.005 (−0.011, 0.021)
G^a^		0.48 (0.47, 0.50)	−0.0002 (−0.018, 0.018)
Other^b^		0.48 (0.46, 0.51)	0.001 (−0.022, 0.024)
LVMI (g/m^2.7^)	704		
R^a^		29.95 (29.40, 30.49)	reference
I^a^		31.13 (29.99, 32.27)	1.18 (−0.08, 2.45)
E^a^		28.99 (26.33, 31.66)	−0.96 (−3.68, 1.76)
J^a^		30.02 (26.90, 33.15)	0.07 (−3.10, 3.25)
G^a^		30.53 (27.49, 33.56)	0.58 (−2.50, 3.66)
Other^b^		33.20 (28.10, 38.30)	3.25 (−1.88, 8.38)

CI, confidence interval; cIMT; carotid intima media thickness, LVMI; Left ventricular mass index, PWV; Pulse wave velocity.

a For details of the specific subgroups contributing to these larger groups see Supplementary Table 1.

b The “other” haplogroup includes (listed in order of frequency): T, Q, H, L, C, N and O. Frequencies of these are also displayed in Supplementary Table 1.

Results of glucose, triglyceride, HDL-c and non-HDL-c were not altered when the observations of participants who ate in the 4 hours before the 15-or 18-year clinics were excluded (Supplementary Figs. 1 and 2). Results for BMI were not altered when the analysis was restricted to participants with 6 or more repeated measures of BMI (Supplementary Fig. 3).

## 4 Discussion

### 4.1 Summary

Understanding the association of common Y chromosomal haplogroups with cardiometabolic risk can provide insights into potential mechanisms underlying cardiometabolic risk in males. In this study, we found no strong evidence that common Y chromosomal haplogroups were associated with cardiometabolic risk factors in males during childhood and adolescence, or with measures of vascular structure and function at age 18. These findings suggest that common variation on the Y chromosome is unlikely to play a role in cardiometabolic risk in males or sex differences in cardiometabolic risk factors which have been shown to exist in early life in several other longitudinal cohorts such as the Bogalusa Heart Study and Project Heartbeat! [[42], [43], [44]].

Our results are comparable with findings from previous studies in adults [[13], [14], [15], [16], [17]]. Charchar and colleagues showed a 50% increased risk of CAD in haplogroup I compared with haplogroup R among 3,233 British men [17]. This association appeared to be independent of conventional cardiovascular risk factors such as BMI, SBP and triglycerides, which our findings support. In addition, Bloomer and colleagues did not find strong evidence of an association between haplogroup I compared with all other haplogroups and cardiometabolic risk factors in 1,988 healthy young men while a Polish study of men aged 20–79 years also found no strong evidence of an association between common Y chromosomal haplogroups (haplogroups R, I, E, N, J, F) and cardiometabolic risk factors [13,15]. Our findings are also in line with a recent Dutch case-control study of males aged ∼70 years which found no difference in Y chromosomal haplogroup distribution (haplogroups R, I, E, G, J) between patients undergoing vascular surgery compared with the general population [14]. Our study replicates these previous findings but in a larger cohort with repeated measures of risk factors across childhood and adolescence.

Our study is the first to examine the association of common Y chromosomal haplogroups with subclinical measures of vascular disease such as PWV, cIMT and LVMI. Whilst we did not observe an association between common Y chromosomal haplogroups and PWV and cIMT, there was an indication that males in haplogroup I had higher LVMI at age 18 years, though confidence intervals spanned the null value. Further analyses of the association of common Y chromosomal haplogroups with these subclinical measures of atherosclerotic risk as this cohort matures may be informative in understanding whether the association with LVMI strengthens with age. Further analyses of these associations will also be of interest as coverage of the Y chromosome in current genotyping arrays improves, given that this is still limited, as compared to coverage available for autosomes. In addition, to date, most association studies of the Y chromosome have been performed in single cohorts, which may be limited in statistical power. Therefore, future association studies of the Y chromosome may benefit from the use of meta-analysis, as has been applied to GWAS of autosomes. This would permit estimates of Y chromosome associations to be combined across multiple studies, allowing for increased power to detect genetic signals on the Y chromosome.

### 4.2 Strengths and limitations

There are several strengths to our study, including its prospective design, availability of repeated measures over time, the ability to examine a range of cardiometabolic risk factors, and the use of multi-level models which take account of clustering of repeated measures within individuals and the correlation between measures over time. Our study is the first to examine the association of common Y chromosomal haplogroups with change over time in cardiometabolic risk factors during childhood and adolescence and with measures of sub-clinical cardiovascular disease. In addition, we have been able to perform subgroup analyses examining haplogroups not investigated in previous analyses such as haplogroups E, J and G. Examining cardiometabolic risk factors during childhood and adolescence reduces the possibility that null associations between common Y chromosomal haplogroups and risk factors are affected by pharmacological treatment, which may attenuate associations in adults. Limitations include combining non-fasting and fasting bloods for risk factors, the availability of measures from birth for only 3 out of the 10 repeatedly measured risk factors, and the inclusion of glucose from NMR spectroscopy at age 7. We acknowledge that assays in cord-blood may not be directly comparable to those measured in serum or plasma later in life. Furthermore, with a period of 9 or more years after the cord blood measures before the next measure of triglycerides, HDL-c and non-HDL-c, there is a strong assumption that these cardiometabolic risk factors change in a linear fashion between birth and age nine. The number of people with measurements of each cardiometabolic risk factor varied, meaning that our analysis samples differed between each cardiometabolic risk factor and were not directly comparable. Loss to follow-up is also a limitation, which may have introduced selection bias. However, we have aimed to minimise potential bias by including all participants with at least a single measure of each risk factor. We have also only been able to examine associations of common Y chromosomal haplogroups with cardiometabolic risk factors in a single study and have not been able to replicate our findings. Therefore, replication of our results in a cohort of similar design is essential to validate our findings. Given that haplogroup I was previously associated with a 50% increased risk of CAD [17], our power calculation showing that we had 80% power to detect at least a difference of 0.2 standard deviations in LVMI between haplogroup I and haplogroup R demonstrates that our analysis is likely to have been sufficient to detect a true difference if one existed. In contrast, as we only had statistical power to detect a difference of 0.6 standard deviations in LVMI for the least common haplogroup (haplogroup G), our analysis may be underpowered to detect smaller effect sizes for this group and our findings require replication in cohorts with greater sample sizes to confirm our findings. In addition, the “other” haplogroups combined category was formed by combining haplogroups for which sample sizes were too small to analyse individually in our study. Therefore, the groups were not analysed together due to phylogenetic relatedness as with categories, R, I, E, J, and G and the results for this haplogroup should be interpreted with caution both due to its heterogeneity and small sample size. We have not adjusted for multiple testing given that our outcomes are not independent of each other and that our overall conclusion that common Y chromosomal haplogroups are not associated with trajectories of cardiometabolic risk in early life would not be altered by doing so. Finally, it is possible that we have not found evidence of an association of common Y chromosomal haplogroups with cardiometabolic measures and vascular outcomes during childhood and adolescence because differences between haplogroups may not become apparent until later in life. Nevertheless, our findings demonstrate that, if haplogroups are associated with cardiometabolic risk in later life, their impact on risk factors or vascular measures is not already evident before the end of adolescence, when the early origins of atherosclerotic originate [[18], [19], [20]].

### 4.3 Conclusion

We found no strong evidence that common Y chromosomal haplogroups are associated with cardiometabolic risk factors in males during childhood and adolescence or with measures of vascular structure and function at age 18.

## Conflicts of interest

The authors declared they do not have anything to disclose regarding conflict of interest with respect to this manuscript.

## Financial support

The MRC Integrative Epidemiology Unit at the University of Bristol is supported by the Medical Research Council and the University of Bristol [MC_UU_12013/6, MC_UU_12013/9]. LMOK is supported by a UK Medical Research Council Population Health Scientist fellowship (MR/M014509/1). LDH and AF are supported by Career Development Awards from the United Kingdom Medical Research Council (grants MR/M020894/1 and MR/M009351/1, respectively). LMOK, LDH, AF, KHW, ELA, DAL, GDS, SR, and ES work in a unit that receives funds from the United Kingdom Medical Research Council (grant MC_UU_12013/5). AH received support from the British Heart Foundation (PG/15/75/31748, CS/15/6/31468, CS/13/1/30327), the Wellcome Trust (086676/7/08/Z), the National Institute for Health Research University College London Hospitals Biomedical Research Centre and works in a unit that receives funds from the United Kingdom Medical Research Council (Programme Code MC_UU_12019/1). All the funding sources had no role in the study design, collection, analysis, or interpretation of the data, writing the manuscript, or the decision to submit the paper for publication.

## Author contributions

LMOK, ES, LDH, and AF designed the study. LMOK performed the analysis and wrote the first draft of the manuscript. KHW performed analyses of vascular measures at age 18. LDH, AF, and ES supervised the analysis of the study. All authors contributed to critical revisions of the analysis and the manuscript.
