# Supplementary material for: Associations of Y chromosomal haplogroups with cardiometabolic risk factors and subclinical vascular measures in males during childhood and adolescence
Source: Atherosclerosis. 2018 Jul;274:94–103. doi: 10.1016/j.atherosclerosis.2018.04.027 (PMC6013646; doi:10.1016/j.atherosclerosis.2018.04.027)
Supplement: Multimedia component 1 [file mmc1.docx]

**Associations of Y chromosomal haplogroups with cardiometabolic risk factors and subclinical vascular measures in males during childhood and adolescence**

**Supplementary Material**

**List of contents**

**Appendix 1:** Details of measurement sources

**Appendix 2:** Details of model selection

**Appendix 3:** Power calculations

**Table 1:** Frequency of haplogroups for CRP and BMI

**Table 2:** Details of power calculations for the association of haplogroup I with each risk factor at age 18

**Table 3:** Details of power calculations for the association of haplogroup G with each risk factor at age 18

**Table 4:** Model details for log BMI trajectories

Table 5: Model details for log fat mass trajectories

Table 6: Model details for lean mass trajectories

Table 7: Model details for SBP, DBP and pulse rate trajectories

**Table 8:** Model details for glucose trajectories

**Table 9:** Model details for log triglyceride trajectories

**Table 10:** Model details for HDL-c trajectories

**Table 11:** Model details for non-HDL-c trajectories

**Table 12:** Model details for log CRP trajectories

**Table 13:** Characteristics of participants by haplogroup

**Table 14:** Mean trajectories of BMI estimated from multilevel models, by haplogroup

**Table 15:** Mean trajectories of fat mass estimated from multilevel models, by haplogroup

**Table 16:** Mean trajectories of lean mass estimated from multilevel models, by haplogroup

**Table 17:** Mean trajectories of blood pressure and pulse rate estimated from multilevel models, by haplogroup

**Table 18:** Mean trajectories of glucose estimated from multilevel models, by haplogroup

**Table 19:** Mean trajectories of triglycerides estimated from multilevel models**,** by haplogroup

**Table 20:** Mean trajectories of cholesterol estimated from multilevel models**,** by haplogroup

Table 21: Mean trajectories of CRP estimated from multilevel models, by haplogroup

**Figure 1** Mean predicted trajectories of glucose from 7-18 years, excluding participants who reported eating before either the 15 or 18-year clinic

**Figure 2** Mean predicted trajectories of log triglycerides, HDL-c and non-HDL-c from birth to 18 years excluding participants who reported eating before either the 15 or 18-year clinic

Figure 3: Mean predicted trajectory of BMI from 1-18 years among participants with 6 or more measures

**Table 1 Frequency of haplogroups for CRP and BMI**

|  | CRP (N = 2,989) | BMI (N=4,693) |
| --- | --- | --- |
| **R** | **2,165 (72.4%)** | **3, 384 (72.1%)** |
| R | 3 (0.1%) | 4 (0.1%) |
| R1 | - | 1 (0.02%) |
| R1a1 | 137 (4.6%) | 208 (4.4%) |
| R1b1 | 286 (9.6%) | 328 (7%) |
| R1b1b | 2 (0.1%) | 2 (0.04%) |
| R1b1b2 | 881 (29.5%) | 1,497 (31.9%) |
| R1b1b2g | 662 (22.2%) | 1,044 (22.3%) |
| R1b1b2h | 188 (6.3%) | 291 (6.2%) |
| R1b1c | - | 1 (0.02%) |
| R2 | 6 (0.2%) | 8 (0.2%) |
| **I** | **550 (18.4%)** | **887 (18.9%)** |
| I | - | 1 (0.02%) |
| I1 | 273 (9.1%) | 445 (9.5%) |
| I1c | 30 (1.0%) | 49 (1.04%) |
| I2 | 96 (3.2%) | 145 (3.1%) |
| I2a2 | 11 (0.4%) | 21 (0.5%) |
| I2b | 140 (4.7%) | 226 (4.8%) |
| **J** | **81 (2.7%)** | **125 (2.7%)** |
| J | 4 (0.1%) | 4 (0.1%) |
| J1 | 12 (0.4%) | 17 (0.4%) |
| J2 | 39 (1.3%) | 67 (1.4%) |
| J2a | 1 (0.3%) | 1 (0.02%) |
| J2a1 | 1 (0.03%) | 2 (0.04%) |
| J2b | 6 (0.2%) | 8 (0.2%) |
| J2b2 | 18 (0.6%) | 26 (0.6%) |
| **E** | **90 (3.0%)** | **140 (3.0%)** |
| E1a | 2 (0.1%) | 2 (0.04%) |
| E1b1 | 4 (0.1%) | 6 (0.1%) |
| E1b1a | 3 (0.1%) | 5 (0.1%) |
| E1b1a7 | - | 1 (0.02%) |
| E1b1a7a | 2 (0.1%) | 4 (0.1% |
| E1b1a8a | - | 1 (0.02%) |
| E1b1b1 | 4 (0.1%) | 4 (0.1%)) |
| E1b1b1a | 9 (0.3%) | 16 (0.3%) |
| E1b1b1a2 | 54 (1.8%) | 79 (1.7%) |
| E1b1b1b2 | 5 (0.2%) | 10 (0.2%) |
| E1b1b1c1 | 6 (0.2%) | 11 (0.2%) |
| E2 | 1 (0.03%) | 1 (0.02%) |
| **G** | **58 (1.9%)** | **86 (1.8%)** |
| G1 | 1 (0.03%) | 2 (0.04%) |
| G2 | 55 (1.8%) | 80 (1.7%) |
| G2C | 2 (0.1%) | 4 (0.1%) |
| **Other** | **45 (1.5%)** | **71 (1.5%)** |
| C | 3 (0.1%) | 5 (0.1%) |
| H | 1 (0.03%) | 1 (0.02%) |
| H1 | 7 (0.2%) | 11 (0.2%) |
| L | 5 (0.2%) | 7 (0.2%) |
| N | - | 1 (0.02%) |
| N1c1 | 3 (0.1%) | 8 (0.2%) |
| O | 1 (0.03%) | 1 (0.02%) |
| O3a | 1 (0.03%) | 3 (0.1%) |
| Q1 | 6 (0.2%) | 7 (0.2%) |
| Q1b | 3 (0.1%) | 5 (0.1%) |
| T | 15 (0.5%) | 22 (0.5%) |
| BMI, body mass index; CRP, c-reactive protein. | | |

**Appendix 1 Details of measurement sources**

*Details on measurement of height and weight at research clinics*

Data from the age 1 year are included in this analysis. We did not include measures before the age of 1 year because the data follow a complex pattern before this age, which cannot be easily modelled. From 1-5 years, measures were available from routine child health clinics for most children and extracted from health visitor records, which form part of standard child care in the UK. Data were also available from research clinic measurements on a random 10% subsample of the cohort. All cohort members were invited to research clinics from the age of 7. Across all ages, parent-reported measures of child height and weight were also available.

At the clinics, crown-heel length for children aged 4-25 months was measured using a Harpenden Neonatometer and from 25 months onwards standing height was measured using a Leicester Height Measure; weight was measured using a Fereday 100kg combined scale (4 month clinic), a Soenhle scale or a Seca scale model 724 (eight month clinic), a Seca 724 or Seca 835 (12 month clinic) and a Seca 835 (18 months onwards). From age 7, all children were invited to annual clinics, at which standing height was measured to the last complete mm using the Harpenden Stadiometer and weight was measured to the nearest 0.1kg using the Tanita Body Fat Analyser (Model TBF 305).

*Details on measurement of blood pressure*

A Dinamap 9301 Vital Signs Monitor (Morton Medical, London) was used at the 7, 9 and 11-year clinics; an Omron MI-5 was used at the 10-year clinic; a Dinamap 8100 Vital Signs Monitor (Morton Medical) was used at the 13-year clinic; and an Omron IntelliSense M6 (Omron Healthcare, Kyoto, Japan) was used at the 15- and 18-year clinics.

*Details on measurement of blood-based biomarkers*

Plasma lipid assays (triglycerides and high-density lipoprotein cholesterol (HDL-c)) were performed by modification of the standard Lipid Research Clinics Protocol using enzymatic reagents for lipid determination. All assay coefficients of variation were <5%. Samples were collected after an overnight fast and were analysed by the hexokinase method.

*Details on measurement of biomarkers using NMR spectroscopy*

A comprehensive profiling of offspring circulating lipids, lipoproteins and metabolites was done by a high-throughput Nuclear Magnetic Resonance (NMR) metabolomics platform, providing a snapshot of offspring serum metabolome at follow-up. (1, 2) At age 7, this was done on fasted blood samples and glucose is included in our analyses. At age 15 and 18, non-fasted bloods assayed using standard clinical chemistry were used.

**Appendix 2 Details of model selection**

Linear splines, fractional polynomials and linear age terms were used in the modelling of trajectories in this paper. Linear splines were used to model all outcomes, except BMI and CRP. Fractional polynomials were used for BMI, due to the complex pattern of change in BMI during childhood and adolescence. This is described elsewhere in detail.(3) Fractional polynomials involve raising age to many combinations of powers, resulting in a wide range of possible curves and offering more flexibility than standard polynomial approaches. CRP was modelled using a linear age term.

Linear spline models were derived by initially examining observed data for each risk factor. We also plotted mean values for each risk factor on each measurement occasion to assist on decisions regarding knot points. We compared observed and predicted measurements for a selection of suitable models for each risk factor. We examined rates of change between time periods in order to examine whether changes between periods were similar or different. In cases where rates of change between two spline periods appeared identical, the fit of models with a reduced number of splines was explored. Systolic blood pressure (SBP) and diastolic blood pressure (DBP) models have been modelled previously and are described elsewhere in detail. (4, 5)

Specific details for each model are described here:

**Fat mass** and **lean mass** were measured on five occasions between 9 and 18 years. Fat mass was log transformed due to skewness of the data. Knots were placed at 13 and 15 years resulting in three periods of change: from 9-13, 13-15 and 15-18. Both models were adjusted for a time varying height co-variate, which was included as a fixed effect.The models took the form of: log fat mass_ij_ or lean mass_ij_ = β_0_ + u_0j_ + (β_1_+ u_1j_ )s_ij1_ + (β_2_+ u_2j_ )s_ij2_ + (β_3_ + u_3j_)s_ij3_ + β_4_ (age-adjusted height covariate)_ij_ + e_ij_, where for person j at measurement occasion I, β_0_ represents the fixed effect coefficient for the average intercept, β_1_ to β_3_ represent fixed effect coefficients for the average linear slopes of each linear spline, s_ij_ represents the specific spline period, β_4_ represents the fixed effect coefficient for the average difference in measurements between individuals of different heights, u_0j_ to u_3j_ indicate person-specific random effects for the intercept and slopes, respectively, and e_ij_ represents the occasion-specific residuals or measurement error, which was allowed to vary with age.

**SBP, DBP** and **pulse rate** were measured at 7 time points from 7 to 18 years. The models for SBP and DBP is described elsewhere in detail. (4, 5) The knots for all models were placed at 12 and 16 resulting in three periods of change: from 7-12, 12-16 and 16-18. All models included a fixed effect to adjust for the use of the use of an Omron MI-5 machine to measure SBP in 10-year clinic, which differed from all other clinics, and a binary time indicator as a level one random effect of age less than or greater than 10 years to account for changing measurement error with age. The models took the form of: SBP_ij_, DBP_ij_ or pulse_ij_ = β_0_ + u_0j_ + (β_1_+ u_1j_)s_ij1_ + (β_2_+ u_2j_)s_ij2_ + (β_3_ + u_3j_)s_ij3_ + β_4_ (machine)_ij_ + e_ij_(age_binary_ij_), where for person j at measurement occasion i, β_0_ represents the fixed effect coefficient for the average intercept, β_1_ to β_3_ represent fixed effect coefficients for the average linear slopes of each linear spline, s_ij_ represents the specific spline period, β_4_ represents the fixed effect coefficient for the average difference in measurements between the machine used at the 10 year clinic compared to the machine used at other clinics, u_0j_ to u_3j_ indicate person-specific random effects for the intercept and slopes, respectively, and e_ij_ represents the occasion-specific residuals or measurement error, which was allowed to vary with age.

**Triglycerides** and **HDL-c** were measured 5 times from birth to 18 years. Triglycerides was log transformed due to the skewness of the data. **Non-HDL-c** was derived by subtracting HDL-c from total cholesterol. Knots for triglycerides and non-HDL-c were placed at 9 and 15 years resulting in 2 periods of change: from birth to 9 years and 9-18. Knots were placed at age 7 and 15 years for HDL-c resulting in two periods of change: from birth to 7 years and 7-18 years. The models took the form of: log triglycerides_ij_, HDL-c_ij_ or non-HDL-c_ij_ = β_0_ + u_0j_ + (β_1_+ u_1j_)s_ij1_ + (β_2_+ u_2j_)s_ij2_ + e_ij_ , where for person j at measurement occasion i, β_0_ represents the fixed effect coefficient for the average intercept, β_1_ and β_2_ represent fixed effect coefficients for the average linear slopes of each linear spline, s_ij_ represents the specific spline period, u_0j_ to u_3j_ indicate person-specific random effects for the intercept and slopes, respectively, and e_ij_ represents the occasion-specific residuals or measurement error.

**Glucose** was measured on 4 occasions from 7 to 18 years. A knot was placed at 15 resulting in 2 periods of change: from 7-15 and 15-18. Due to few available repeated measures of glucose, we modelled the person-specific random effects as a single linear slope rather than a function of the splines. This allowed person-specific variation from the average trajectory but under the assumption that person-specific deviation from the mean trajectory was constant over time. The models took the form of: glucose_ij_ = β_0_ + u_0j_ + (β_1_)s_ij1_ + (β_2_)s_ij2_ + u_1j_*age + e_ij_ , where for person j at measurement occasion i, β_0_ represents the fixed effect coefficient for the average intercept, β_1_ and β_2_ represent fixed effect coefficients for the average linear slopes of each linear spline, s_ij_ represents the specific spline period, u_0j_ to u_1j_ indicate person-specific random effects for the intercept and slope, respectively, and e_ij_ represents the occasion-specific residuals or measurement error.

**BMI** has been modelled previously using fractional polynomials and is described elsewhere. (3) Briefly, BMI was log transformed due to skewness of the data and fractional polynomials were used where age was raised to various combinations of powers (each of the following single powers, plus each combination of two powers: 0.5, 1, 2, 3, -0.5, -1, -2 and natural log), from which we selected the best fitting curve (the one with the lowest likelihood value). The resulting curve contained three age terms including log age, log age* age and log age *age^2.^ To account for the likely reduced accuracy of parent-reported measurements, a binary indicator of measurement source (research clinic or health records versus parent-report) was included as a fixed effect. The variance of measurement occasion-level residuals (the difference between observed and predicted measurements) was allowed to vary with age for log BMI. To allow comparability of log BMI with fat and lean mass, predicted BMI corresponding to the knot points for lean/fat mass (9-13, 13-15 and 15-18) were calculated. The models took the form of: log BMI_ij_ = (β_0_+u_0j_+e_0ij_) + (β_1_+u_1j_)(ln(age)_ij_) + (β_2_+u_2j_)(age*ln(age)_ij_) + (β_3_+u_3j_)(age^2^*ln(age)_ij_) + (β_4_+e_1ij_)(measurement_source_ij_) + e_ij_(age_months_ij_), where for person j at measurement occasion i, β’s represent fixed effect coefficients, u_0j_ to u_3j_ indicate person-specific random effects for the intercept and linear, quadratic and cubic age terms, respectively, and e_1_ represents the occasion-specific residuals or measurement error, which was allowed to vary with age and according to measurement source.

**CRP** was measured on 3 occasions from 9 to 18 years and modelled using a linear age term. CRP was log transformed due to skewness of the data. A knot was placed at 15 resulting in 2 periods of change: from 9-15 and 15-18. The models for CRP took the form of: log CRP _ij_ = β_0_ + u_0j_ + (β_1_+ u_1j_) age_ij1_ + e_ij_(age_ij_), where for person j at measurement occasion i, β_0_ represents the fixed effect coefficient for the average intercept, β_1_ represents fixed effect coefficient for the average linear slope, u_0j_ and u_1j_ indicate person-specific random effects for the intercept and slopes, respectively, and e_ij_ represents the occasion-specific residuals or measurement error, which was allowed to vary with age.

Observed and predicted measurements for each model are shown in Table 2-10.

**Appendix 3 Power Calculations**

We performed power calculations for all risk factors at age 18 in our analysis. Previous publications have identified haplgroup I as having a 50% greater coronary artery disease (CAD) risk compared with haplogroup R. (5) This group was our a priori comparison of interest in the analysis given that one of our aims was to examine if associations observed in adults are already present in early life.  We therefore performed a power calculation for the association of haplogroup I with each outcome at age 18 (Table 2). We also performed a power calculation for the association of the smallest group in our analysis (haplogroup G) with each outcome at age 18. All calculations were based on 80% power and 0.05 significance level and effect sizes are expressed as standard deviations and as absolute differences. We found that for most risk factors at age 18, we had 80% power at an alpha value of 0.05 to detect an effect size difference of 0.2 standard deviations (SD) between haplogroup I and haplogroup R. We found that for most risk factors at age 18, we had 80% power at an alpha value of 0.05 to detect an effect size difference of 0.5 or 0.6 SDs between haplogroup G and haplogroup R.

| Risk factor | N in haplogroup R | N in haplogroup I | Power | Alpha | Effect size (SD) | Absolute effect size |
| --- | --- | --- | --- | --- | --- | --- |
| BMI | 1,316 | 324 | 80% | 0.05 | 0.2 | 0.8 kg/m^2^ |
| Fat | 1,323 | 323 | 80% | 0.05 | 0.2 | 1.9 kg |
| Lean | 1,323 | 323 | 80% | 0.05 | 0.2 | 1.3 kg |
| SBP | 1,266 | 320 | 80% | 0.05 | 0.2 | 1.8 mmHg |
| DBP | 1,266 | 320 | 80% | 0.05 | 0.2 | 1.2 mmHg |
| Pulse | 1,266 | 320 | 80% | 0.05 | 0.2 | 1.8 bpm |
| Glucose | 1,010 | 245 | 80% | 0.05 | 0.2 | 0.07 mmol/l |
| Triglyceride | 1,019 | 248 | 80% | 0.05 | 0.2 | 0.06 mmol/l |
| HDL-c | 1,019 | 248 | 80% | 0.05 | 0.2 | 0.05 mmol/l |
| Non-HDL-c | 1,019 | 248 | 80% | 0.05 | 0.2 | 0.1 mmol/l |
| CRP | 1,029 | 249 | 80% | 0.05 | 0.2 | 0.3 mg/l |
| PWV | 924 | 226 | 80% | 0.05 | 0.2 | 0.14 m/s |
| cIMT | 1,127 | 280 | 80% | 0.05 | 0.2 | 0.008 mm |
| LVMI | 523 | 120 | 80% | 0.05 | 0.3 | 1.8 g/m^2.7^ |

**Table 2 Details of power calculations for the association of haplogroup I with each risk factor at age 18**

bpm, beats per minute; cIMT, carotid intima media thickness; CRP, c-reactive protein; DBP, diastolic blood pressure; HDL-c, high density lipoprotein cholesterol; LVMI, left ventricular mass index; PWV, pulse wave velocity; SBP, systolic blood pressure.

**Table 3 Details of power calculations for the association of haplogroup G with each risk factor at age 18**

| Risk factor | N in haplogroup R | N in haplogroup I | Power | Alpha | Effect size (SD) | Absolute effect size |
| --- | --- | --- | --- | --- | --- | --- |
| BMI | 1,316 | 32 | 80% | 0.05 | 0.5 | 1.9 kg/m^2^ |
| Fat | 1,323 | 33 | 80% | 0.05 | 0.5 | 4.8 kg |
| Lean | 1,323 | 33 | 80% | 0.05 | 0.5 | 3.1 kg |
| SBP | 1,266 | 31 | 80% | 0.05 | 0.5 | 4.5 mmHg |
| DBP | 1,266 | 31 | 80% | 0.05 | 0.5 | 3 mmHg |
| Pulse | 1,266 | 31 | 80% | 0.05 | 0.5 | 4.5 bpm |
| Glucose | 1,010 | 28 | 80% | 0.05 | 0.5 | 0.2 mmol/l |
| Triglyceride | 1,019 | 28 | 80% | 0.05 | 0.6 | 0.2 mmol/l |
| HDL-c | 1,019 | 28 | 80% | 0.05 | 0.6 | 0.15 mmol/l |
| Non-HDL-c | 1,019 | 28 | 80% | 0.05 | 0.6 | 0.3 mmol/l |
| CRP | 1,029 | 29 | 80% | 0.05 | 0.6 | 0.9 mg/l |
| PWV | 924 | 24 | 80% | 0.05 | 0.6 | 0.42 m/s |
| cIMT | 1127 | 26 | 80% | 0.05 | 0.6 | 0.02 mm |
| LVMI | 523 | 17 | 80% | 0.05 | 0.7 | 4.2 g/m^2.7^ |

bpm, beats per minute; cIMT, carotid intima media thickness; CRP, c-reactive protein; DBP, diastolic blood pressure; HDL-c, high density lipoprotein cholesterol; LVMI, left ventricular mass index; PWV, pulse wave velocity; SBP, systolic blood pressure.

Table4 Model details for log BMI trajectories

|  | No of contributing individuals | | Assessment of model fit | | | |
| --- | --- | --- | --- | --- | --- | --- |
|  | Total number of observations | Number of individuals with 1 measure | Mean observed  BMI, ln(kg/m^2^) (SD) ^a^ | Mean predicted  BMI, ln(kg/m^2^) (SD) ^a^ | Mean difference (observed – predicted), ln(kg/m^2^) ^a^ | 95% level of agreement between observed and predicted, ln(kg/m^2^) ^a^ |
| Overall | 45,316 | 4,693 |  |  |  |  |
| 1-3 years | 7,735 | 3,709 | 2.84 (0.09) | 2.84 (0.06) | 0.01 | -0.10 to 0.09 |
| 3-7 years | 10,068 | 4,044 | 2.78 (0.10) | 2.77 (0.07) | 0.01 | -0.13 to 0.14 |
| 7-9 years | 6,071 | 3,566 | 2.79 (0.12) | 2.80 (0.11) | -0.01 | -0.07 to 0.06 |
| 9-11 years | 6,851 | 3,469 | 2.86 (0.15) | 2.86 (0.14) | 0.00 | -0.07 to 0.07 |
| 11-13 years | 5,469 | 3,185 | 2.93 (0.17) | 2.92 (0.15) | 0.01 | -0.07 to 0.09 |
| 13-15 years | 5,007 | 3,079 | 2.97 (0.16) | 2.97 (0.16) | 0.01 | -0.11 to 0.11 |
| 15-18 years | 4,115 | 2,525 | 3.06 (0.16) | 3.07 (0.15) | -0.01 | -0.08 to 0.07 |

^a^ BMI is natural log transformed. All values are in log form.

Table 5 Model details for log fat mass trajectories

|  | No of contributing individuals | | Assessment of model fit | | | |
| --- | --- | --- | --- | --- | --- | --- |
|  | Total number of observations | Number of individuals with 1 measure | Mean observed fat mass, ln(kg) (SD) ^a^ | Mean predicted fat mass, ln(kg) (SD) ^a^ | Mean difference (observed – predicted), ln(kg) ^a^ | 95% level of agreement between observed and predicted, ln(kg) ^a^ |
| Overall | 12,554 | 3,624 |  |  |  |  |
| 9 years | 3,090 | 3,090 | 1.80 (0.60) | 1.81 (0.55) | -0.01 | -0.22 to 0.19 |
| 9-13 years | 6,034 | 3,457 | 1.97 (0.62) | 1.96 (0.58) | 0.01 | -0.22 to 0.24 |
| 13-15 years | 2,576 | 2,565 | 2.19 (0.62) | 2.23 (0.58) | -0.03 | -0.33 to 0.27 |
| 15-18 years | 3,944 | 2,443 | 2.31 (0.63) | 2.30 (0.60) | 0.01 | -0.22 to 0.23 |
|  |  |  |  |  |  |  |

^a^ Fat mass is natural log transformed. All values are in log form.

Table 6 Model details for lean mass trajectories

|  | No of contributing individuals | | Assessment of model fit | | | |
| --- | --- | --- | --- | --- | --- | --- |
|  | Total number of observations | Number of individuals with 1 measure | Mean observed lean mass, kg (SD) | Mean predicted lean mass, kg (SD) | Mean difference (observed – predicted), kg | 95% level of agreement between observed and predicted, kg |
| Overall | 12,573 | 3,627 |  |  |  |  |
| 9 years | 3,098 | 3,098 | 25.52 (2.95) | 25.56 (2.40) | -0.04 | -3.04 to 2.96 |
| 9-13 years | 6,043 | 3,461 | 27.78 (4.28) | 27.75 (3.98) | 0.03 | -2.73 to 2.79 |
| 13-15 years | 2,578 | 2,567 | 40.91 (7.15) | 41.05 (6.26) | -0.15 | -3.62 to 3.32 |
| 15-18 years | 3,952 | 2,447 | 52.19 (7.05) | 52.14 (6.83) | 0.05 | -2.22 to 2.32 |
|  |  |  |  |  |  |  |

Table 7 Model details for SBP, DBP and pulse rate trajectories

|  | No of contributing individuals | | Assessment of model fit | | | |
| --- | --- | --- | --- | --- | --- | --- |
|  | Total number of observations | Number of individuals with 1 measure | Mean observed SBP, DBP or pulse rate (SD) ^a^ | Mean predicted SBP, DBP or pulse rate (SD) ^a^ | Mean difference (observed – predicted) ^a^ | 95% level of agreement between observed and predicted ^a^ |
| SBP |  |  |  |  |  |  |
| Overall | 19,332 | 3,981 |  |  |  |  |
| 7 years | 3,417 | 3,417 | 98.68 (9.09) | 98.60 (5.40) | 0.08 | -10.54 to 10.69 |
| 7-12 years | 12,375 | 3,894 | 102.26 (9.35) | 102.38 (6.13) | -0.12 | -11.52 to 11.28 |
| 12-16 years | 5,104 | 3,024 | 116.93 (12.40) | 116.53 (9.66) | 0.40 | -11.48 to 12.29 |
| 16-18 years | 1,853 | 1,801 | 122.26 (9.41) | 122.56 (5.91) | -0.30 | -12.09 to 11.50 |
|  |  |  |  |  |  |  |
| DBP |  |  |  |  |  |  |
| Overall | 19,332 | 3,981 |  |  |  |  |
| 7 years | 3,417 | 3,417 | 55.90 (6.58) | 56.16 (3.38) | -0.26 | -8.92 to 8.40 |
| 7-12 years | 12,375 | 3,894 | 57.56 (6.86) | 57.14 (3.58) | 0.42 | -9.47 to 10.30 |
| 12-16 years | 5,104 | 3,024 | 61.21 (10.04) | 62.11 (5.57) | -0.90 | -12.56 to 10.76 |
| 16-18 years | 1,853 | 1,801 | 63.39 (6.23) | 63.75 (3.73) | -0.36 | -12.81 to 12.10 |
|  |  |  |  |  |  |  |
| Pulse rate |  |  |  |  |  |  |
| Overall | 19,332 | 3,981 |  |  |  |  |
| 7 years | 3,417 | 3,417 | 81.66 (10.58) | 81.47 (6.05) | 0.19 | -12.29 to 12.68 |
| 7-12 years | 12,375 | 3,894 | 75.58 (11.47) | 75.63 (7.76) | -0.05 | -13.33 to 13.23 |
| 12-16 years | 5,104 | 3,024 | 71.95 (11.10) | 71.73 (6.77) | 0.22 | -13.07 to 13.51 |
| 16-18 years | 1,853 | 1,801 | 62.92 (9.42) | 63.18 (5.41) | -0.26 | -14.50 to 13.97 |
|  |  |  |  |  |  |  |
|  |  |  |  |  |  |  |

DBP, diastolic blood pressure; SBP, systolic blood pressure.

^a^units are presented in mmHg for SBP and DBP and bpm for pulse rate

**Table 8 Model details for glucose trajectories**

|  | No of contributing individuals | | Assessment of model fit | | | |
| --- | --- | --- | --- | --- | --- | --- |
|  | Total number of observations | Number of individuals with 1 measure | Mean observed glucose, mmol/l (SD) | Mean predicted glucose, mmol/l (SD) | Mean difference (observed – predicted), mmol/l | 95% level of agreement between observed and predicted, mmol/l |
| Overall | 5,484 | 3,060 |  |  |  |  |
| 7 years | 2,136 | 2,136 | 4.37 (0.41) | 4.39 (0.28) | -0.03 | -0.32 to 0.27 |
| 7-15 years | 2,570 | 2,307 | 4.47 (0.46) | 4.46 (0.32) | 0.01 | -0.36 to 0.37 |
| 15-18 years | 2,914 | 1,952 | 5.22 (0.36) | 5.23 (0.14) | -0.01 | -0.67 to 0.64 |
|  |  |  |  |  |  |  |

Table 9 Model details for log triglycerides trajectories

|  | No of contributing individuals | | Assessment of model fit | | | |
| --- | --- | --- | --- | --- | --- | --- |
|  | Total number of observations | Number of individuals with 1 measure | Mean observed triglycerides, ln(mmol/l)  (SD) ^a^ | Mean predicted triglycerides, ln(mmol/l)  (SD) ^a^ | Mean difference (observed – predicted), ln(mmol/l) ^a^ | 95% level of agreement between observed and predicted, ln(mmol/l) ^a^ |
| Overall | 9,665 | 4,192 |  |  |  |  |
| Birth | 1,757 | 1,757 | -0.68 (0.45) | -0.68 (0.21) | 0.01 | -0.40 to 0.41 |
| 0-9 years | 4,270 | 3,487 | -0.33 (0.53) | -0.33 (0.35) | 0.01 | -0.38 to 0.38 |
| 9-18 years | 5,395 | 2,978 | -0.16 (0.42) | -0.15 (0.22) | 0.01 | -0.34 to 0.34 |

ln(trig), natural log of triglyceride.

^a^ Triglycerides is natural log transformed. All values are in log form.

Table 10 Model details for HDL-c trajectories

|  | No of contributing individuals | | Assessment of model fit | | | |
| --- | --- | --- | --- | --- | --- | --- |
|  | Total number of observations | Number of individuals with 1 measure | Mean observed HDL-c, mmol/l (SD) | Mean predicted HDL-c, mmol/l (SD) | Mean difference (observed – predicted), mmol/l | 95% level of agreement between observed and predicted, mmol/l |
| Overall | 9,692 | 4,189 |  |  |  |  |
| Birth | 1,740 | 1,740 | 0.51 (0.23) | 0.51 (0.11) | 0.01 | -0.23 to 0.23 |
| 0-7 years | 1,741 | 1,741 | 0.51 (0.23) | 0.51 (0.12) | 0.01 | -0.23 to 0.23 |
| 7-18 years | 7,951 | 3,598 | 1.38 (0.32) | 1.38 (0.26) | 0.01 | -0.25 to 0.25 |

HDL-c, high density lipoprotein cholesterol.

Table 11 Model details for non-HDL-c trajectories

|  | No of contributing individuals | | Assessment of model fit | | | |
| --- | --- | --- | --- | --- | --- | --- |
|  | Total number of observations | Number of individuals with 1 measure | Mean observed non-HDL-c, mmol/l (SD) | Mean predicted non-HDL-c, mmol/l (SD) | Mean difference (observed – predicted), mmol/l | 95% level of agreement between observed and predicted, mmol/l |
| Overall | 9,664 | 4,186 |  |  |  |  |
| Birth | 1,709 | 1,709 | 1.18 (0.53) | 1.21 (0.28) | 0.02 | -0.53 to 0.57 |
| 0-9 years | 4,229 | 3,471 | 2.13 (0.97) | 2.08 (0.81) | 0.01 | -0.71 to 0.71 |
| 9-18 years | 5,435 | 2,993 | 2.55 (0.64) | 2.59 (0.52) | 0.02 | -0.88 to 0.92 |
|  |  |  |  |  |  |  |

Non-HDL-c, non-high density lipoprotein cholesterol.

Table 12 Model details for log CRP trajectories

|  | No of contributing individuals | | Assessment of model fit | | | |
| --- | --- | --- | --- | --- | --- | --- |
|  | Total number of observations | Number of individuals with 1 measure | Mean observed CRP, ln(mgl/l)(SD) ^a^ | Mean predicted CRP, ln(mg/l) (SD) ^a^ | Mean difference (observed – predicted), ln(mg/)l ^a^ | 95% level of agreement between observed and predicted, ln(mg/l )^a^ |
| Overall | 2989 |  |  |  |  | 2989 |
| Age 9 | 2423 | -1.53 (1.21) | -1.51 (0.54) | -0.02 | -1.44 to 1.40 | 2423 |
| Age 15 | 1575 | -0.70 (1.10) | -0.78 (0.44) | 0.09 | -1.50 to 1.67 | 1575 |
| Age 18 | 1399 | -0.60 (1.06) | -0.53 (0.40) | -0.07 | -1.58 to 1.44 | 1399 |

CRP, C - reactive protein.

^a^ CRP is natural log transformed. All values are in log form.

**Table 13 Characteristics of participants by haplogroup**

|  | **Haplogroup R**  **N=2,757** | **Haplogroup I**  **n=726** | **Other haplogroups** ^a^  **N=329** | **P value for comparison** ^b^ |
| --- | --- | --- | --- | --- |
|  | n (%) | n (%) | n (%) |  |
| **Maternal marital status (N=3,812)** |  |  |  |  |
| Never married | 346(12.5) | 79(10.9) | 50(15.2) | 0.319 |
| Widowed | 3(0.1) | 2(0.3) | 0(0.0) |  |
| Divorced | 107(3.9) | 27(3.7) | 8(2.4) |  |
| Separated | 33(1.2) | 7(1.0) | 0(0.0) |  |
| 1^st^ Marriage | 2,070(75.1) | 554(76.3) | 249(75.7) |  |
| Marriage 2 or 3 | 198(7.2) | 57(7.9) | 22(6.7) |  |
| **Household social class (N=3,812)** |  |  |  |  |
| Professional | 424(15.4) | 116(16.0) | 49(14.9) | 0.587 |
| Managerial & Technical | 1,196(43.4) | 293(40.4) | 150(45.6) |  |
| Non-Manual | 705(25.6) | 192(26.4) | 83(25.2) |  |
| Manual | 306(11.1) | 88(12.1) | 39(11.9) |  |
| Part Skilled & Unskilled | 126(4.6) | 37(5.1) | 8(2.4) |  |
| **Maternal education (N=3,812)** |  |  |  |  |
| Less than O level | 647(23.5) | 176(24.2) | 73(22.2) | 0.865 |
| O level | 986(35.8) | 254(35.0) | 124(37.7) |  |
| A level | 703(25.5) | 183(25.2) | 75(22.8) |  |
| Degree or above | 421(15.3) | 113(15.6) | 57(17.3) |  |
| **Partners highest educational qualification (N=3,812)** |  |  |  |  |
| Less than O level | 762(27.6) | 222(30.6) | 95(28.9) | 0.381 |
| O level | 626(22.7) | 141(19.4) | 81(24.6) |  |
| A level | 781(28.3) | 203(28.0) | 89(27.1) |  |
| Degree or Above | 588(21.3) | 160(22.0) | 64(19.5) |  |
| **Maternal smoking during pregnancy (N=3,812)** |  |  |  |  |
| Yes | 2,229(80.8) | 584(80.4) | 266(80.9) | 0.969 |
| No | 528(19.2) | 142(19.6) | 63(19.1) |  |

^a^ Other includes E, J, G T, Q, H, L, C, N, O in this table only, solely for the purposes of comparing baseline characteristics of mothers of participants across haplogroups.

^b^ p value is for the difference in proportions for categorical variables from *χ*² test.

**Table 14 Mean trajectories of BMI estimated from multilevel models, by haplogroup**

|  | **Mean log BMI trajectory (95% CI) in haplogroup R (kg/m**^2^ **) (reference)** ^a^ | **Mean difference in BMI trajectory (95% CI) comparing haplogroup I with haplogroup R (% difference in kg/m**^2^**)** ^b^ | **Mean difference in log BMI trajectory (95% CI) comparing haplogroup E with haplogroup R (% difference in kg/m**^2^**)** ^b^ | **Mean difference in log BMI trajectory (95% CI) comparing haplogroup J with haplogroup R (% difference in kg/m**^2^**)** ^b^ | **Mean difference in log BMI trajectory (95% CI) comparing haplogroup G with haplogroup R (% difference in kg/m**^2^**)** ^b^ | **Mean difference in log BMI trajectory (95% CI) comparing all other haplogroups combined with haplogroup R (% difference in kg/m**^2^**)** ^b^ |
| --- | --- | --- | --- | --- | --- | --- |
|  |  |  |  |  |  |  |
| **BMI** |  |  |  |  |  |  |
| Age 1yr | 3.34 (3.32,3.36) | -0.12 (-4.59,4.35) | 2.51 (-8.29,13.31) | -2.76 (-12.96,7.45) | 4.4 (-8.73,17.43) | -4.92 (-18.51,8.66) |
| Age 3yr | 2.80 (2.80,2.80) | -0.19 (-0.79,0.41) | -0.62 (-2.02,0.78) | 1.36 (-0.11,2.83) | 0.3 (-1.48,2.01) | -0.83 (-2.76,1.10) |
| Age 7yr | 2.76 (2.76,2.77) | -0.19 (-0.91,0.54) | -0.99 (-2.64,0.67) | 1.40 (-0.35,3.16) | 0.7 (-1.38,2.79) | -0.74 (-3.03,1.55) |
| Age 9yr | 2.80 (2.80,2.81) | -0.11 (-1.01,0.79) | -0.97 (-3.01,1.08) | 1.25 (-0.92,3.42) | 1.1 (-1.48,3.75) | -1.01 (-3.81,1.80) |
| Age 11yr | 2.86 (2.85,2.86) | 0.03 (-1.06,1.12) | -0.96 (-3.43,1.51) | 1.22 (-1.41,3.84) | 1.5 (-1.72,4.64) | -1.28 (-4.65,2.10) |
| Age 13yr | 2.92 (2.91,2.92) | 0.23 (-0.99,1.46) | -1.00 (-3.77,1.76) | 1.36 (-1.59,4.31) | 1.6 (-1.94,5.20) | -1.49 (-5.27,2.28) |
| Age 15yr | 2.99 (2.98,2.99) | 0.50 (-0.79,1.80) | -1.11 (-4.00,1.78) | 1.72 (-1.40,4.84) | 1.6 (-2.15,5.35) | -1.63 (-5.59,2.33) |
| Age 18yr | 3.10 (3.09,3.10) | 1.04 (-0.36,2.44) | -1.43 (-4.51,1.65) | 2.72 (-0.72,6.15) | 1.1 (-2.90,5.15) | -1.65 (-5.95,2.66) |

CI, confidence interval; yr, year.

^a^ BMI is presented in the natural log and values represent the mean predicted natural log of BMI at each age shown.

^b^ Differences at each age are back transformed from the log scale and are interpreted as the percentage difference in the mean level at each age comparing each category with haplogroup R. For details of the specific subgroups contributing to these larger groups see Supplementary Table 1. The “other” haplogroup includes (listed in order of frequency): T, Q, H, L, C, N and O.

**Table 15 Mean trajectories of fat mass estimated from multilevel models, by haplogroup**

|  | **Mean log fat mass trajectory (95% CI) in haplogroup R (kg or kg/yr** **) (reference)** ^a^ | **Mean difference in fat mass trajectory (95% CI) comparing haplogroup I with haplogroup R (% or %/yr)** ^b^ | **Mean difference in fat mass trajectory (95% CI) comparing haplogroup E with haplogroup R (% or %/yr)** ^b^ | **Mean difference in fat mass trajectory (95% CI) comparing haplogroup J with haplogroup (% or %/yr)** ^b^ | **Mean difference in fat mass trajectory (95% CI) comparing haplogroup G with haplogroup R (% or %/yr)** ^b^ | **Mean difference in fat mass trajectory (95% CI) comparing all other haplogroups combined with haplogroup R (% or %/yr)** ^b^ |
| --- | --- | --- | --- | --- | --- | --- |
|  |  |  |  |  |  |  |
|  |  |  |  |  |  |  |
| Age 9yr (kg) or (%) | 1.76 (1.73,1.79) | -0.92 (-6.46,4.61) | -4.00 (-16.27,8.27) | 7.21 (-6.89,21.30) | 6.5 (-10.32,23.37) | -3.48 (-20.02,13.06) |
| Change 9-13yr (kg/yr) or (%/yr) | 0.13 (0.12,0.14) | 0.18 (-1.04,1.40) | -1.47 (-4.14,1.20) | -0.31 (-3.08,2.46) | 0.9 (-2.54,4.39) | 1.37 (-2.49,5.24) |
| Change 13-15yr (kg/yr) or (%/yr) | -0.06 (-0.07,-0.05) | 0.18 (-2.03,2.39) | 2.27 (-2.74,7.28) | 1.94 (-3.24,7.12) | -1.8 (-7.73,4.14) | 1.28 (-5.78,8.34) |
| Change 15-18yr (kg/yr) or (%/yr) | 0.10 (0.09,0.11) | 0.47 (-1.37,2.30) | -1.57 (-5.70,2.55) | -0.87 (-5.23,3.50) | -0.7 (-5.75,4.42) | -4.39 (-9.97,1.18) |
| Age 18yr (kg) or (%) | 2.43 (2.40,2.46) | 1.55 (-5.11,8.22) | -3.56 (-20.81,13.69) | 7.17 (-9.95,24.29) | 4.5 (-14.99,23.99) | -8.64 (-27.73,10.46) |

CI, confidence interval; kg/yr, kilograms per year; %/yr, percentage per year.

^a^ Fat mass was transformed using the natural log. All predicted mean values (kg) and rates of change per year (kg/yr) are on the log scale

^b^ The difference between haplogroups is back transformed from the log scale for ease of interpretation and is interpreted as the percentage difference in the mean level comparing each category with haplogroup R or percentage difference in change per year (%/yr) comparing each category with haplogroup R. For details of the specific subgroups contributing to these larger groups see Supplementary Table 1. The “other” haplogroup includes (listed in order of frequency): T, Q, H, L, C, N and O.

**Table 16 Mean trajectories of lean mass estimated from multilevel models, by haplogroup**

|  | **Mean trajectory (95% CI) in haplogroup R (reference)** | **Mean difference in trajectory (95% CI) comparing haplogroup I with haplogroup R** | **Mean difference in trajectory (95% CI) comparing haplogroup E with haplogroup R** | **Mean difference in trajectory (95% CI) comparing haplogroup J with haplogroup R** | **Mean difference in trajectory (95% CI) comparing haplogroup G with haplogroup R** | **Mean difference in trajectory (95% CI) comparing all other haplogroups combined with haplogroup R** |
| --- | --- | --- | --- | --- | --- | --- |
|  |  |  |  |  |  |  |
|  |  |  |  |  |  |  |
| Age 9yr (kg) | 23.98 (23.84,24.12) | -0.10 (-0.36,0.17) | 0.07 (-0.52,0.66) | 0.69 (0.08,1.30) | 0.86 (0.11,1.61) | -0.57 (-1.36,0.23) |
| Change 9-13yr (kg/yr) | 2.33 (2.26,2.40) | -0.06 (-0.20,0.08) | -0.06 (-0.36,0.25) | 0.08 (-0.23,0.40) | -0.11 (-0.49,0.27) | -0.43 (-0.86,0.00) |
| Change 13-15yr (kg/yr) | 7.65 (7.54,7.75) | 0.05 (-0.18,0.28) | -0.25 (-0.76,0.27) | -0.24 (-0.78,0.30) | 0.45 (-0.19,1.09) | -0.37 (-1.10,0.36) |
| Change 15-18yr (kg/yr) | 2.53 (2.43,2.62) | -0.18 (-0.40,0.03) | 0.32 (-0.16,0.81) | 0.22 (-0.29,0.72) | -0.004 (-0.60,0.59) | 0.21 (-0.46,0.88) |
| Age 18yr (kg) | 56.19 (55.90,56.48) | -0.79 (-1.43,-0.14) | 0.32 (-1.45,2.09) | 1.20 (-0.37,2.76) | 1.30 (-0.53,3.14) | -2.39 (-4.44,-0.34) |

kg/yr, kilograms per year. For details of the specific subgroups contributing to these larger groups see Table 1. The “other” haplogroup includes (listed in order of frequency): T, Q, H, L, C, N and O.

Table 17 Mean trajectories of blood pressure and pulse rate estimated from multilevel models, by haplogroup

|  | Mean trajectory (95% CI) in haplogroup R (reference) | Mean difference in trajectory (95% CI) comparing haplogroup I with haplogroup R | | Mean difference in trajectory (95% CI) comparing haplogroup E with haplogroup R | Mean difference in trajectory (95% CI) comparing haplogroup J with haplogroup R | Mean difference in trajectory (95% CI) comparing haplogroup G with haplogroup R | Mean difference in trajectory (95% CI) comparing all other haplogroups with haplogroup R |
| --- | --- | --- | --- | --- | --- | --- | --- |
|  |  |  | |  |  |  |  |
| SBP |  |  | |  |  |  |  |
| Age 7yr (mmHg) | 97.87 (97.51,98.23) | -0.55 (-1.35,0.25) | -0.35 (-2.17,1.47) | | 2.07 (0.19,3.96) | 0.37 (-1.87,2.60) | 0.60 (-1.90,3.11) |
| Change 7-12yr (mmHg/yr) | 1.60 (1.51,1.69) | 0.26 (0.07,0.45) | 0.06 (-0.37,0.50) | | -0.20 (-0.65,0.25) | -0.19 (-0.71,0.33) | -0.29 (-0.90,0.33) |
| Change 12-16yr (mmHg/yr) | 5.91 (5.76,6.06) | -0.30 (-0.62,0.02) | -0.29 (-0.99,0.42) | | 0.45 (-0.33,1.23) | -0.24 (-1.14,0.66) | -0.37 (-1.38,0.65) |
| Change 16-18yr (mmHg/yr) | -4.04 (-4.38,-3.69) | 0.41 (-0.35,1.18) | 1.38 (-0.29,3.06) | | -1.45 (-3.36,0.47) | 0.14 (-2.11,2.39) | -0.23 (-2.64,2.19) |
| Age 18yr (mmHg) | 121.43 (120.92,121.95) | 0.39 (-0.75,1.53) | 2.56 (-0.76,5.89) | | -0.02 (-2.87,2.83) | -1.28 (-4.70,2.13) | -2.75 (-6.38,0.88) |
| DBP |  |  |  | |  |  |  |
| Age 7yr (mmHg) | 56.17 (55.91,56.44) | -0.26 (-0.84,0.33) | 0.24 (-1.10,1.58) | | 0.56 (-0.83,1.95) | 1.22 (-0.42,2.85) | -0.61 (-2.45,1.23) |
| Change 7-12yr (mmHg/yr) | 0.15 (0.08,0.22) | 0.04 (-0.11,0.19) | -0.04 (-0.39,0.30) | | -0.003 (-0.36,0.35) | -0.29 (-0.70,0.12) | 0.03 (-0.46,0.51) |
| Change 12-16yr (mmHg/yr) | 2.85 (2.71,2.99) | -0.10 (-0.40,0.21) | 0.01 (-0.65,0.67) | | 0.10 (-0.63,0.84) | 0.88 (0.03,1.73) | 0.11 (-0.85,1.07) |
| Change 16-18yr (mmHg/yr) | -2.65 (-2.96,-2.34) | 0.40 (-0.30,1.09) | 0.20 (-1.30,1.69) | | -0.76 (-2.51,0.99) | -1.74 (-3.78,0.29) | -0.76 (-2.95,1.44) |
| Age 18yr (mmHg) | 63.02 (62.65,63.39) | 0.38 (-0.45,1.20) | 0.88 (-1.58,3.33) | | -0.57 (-2.65,1.51) | -0.20 (-2.70,2.29) | -1.55 (-4.14,1.05) |
| Pulse rate |  |  | |  |  |  |  |
| Age 7yr (bpm) | 82.49 (82.06,82.92) | -0.16 (-1.11,0.78) | | 0.37 (-1.77,2.52) | 0.22 (-2.01,2.44) | 0.03 (-2.61,2.66) | 3.02 (0.07,5.97) |
| Change 7-12yr (bpm/yr) | -1.84 (-1.94,-1.74) | -0.10 (-0.33,0.12) | | -0.25 (-0.77,0.27) | -0.12 (-0.67,0.42) | -0.25 (-0.88,0.38) | -0.09 (-0.82,0.65) |
| Change 12-16yr (bpm/yr) | -0.77 (-0.93,-0.61) | 0.06 (-0.29,0.41) | | 0.83 (0.06,1.59) | 0.51 (-0.33,1.36) | 0.19 (-0.79,1.17) | -0.20 (-1.31,0.91) |
| Change 16-18yr (bpm/yr) | -4.23 (-4.59,-3.87) | 0.11 (-0.68,0.91) | | -0.16 (-1.91,1.58) | -0.75 (-2.76,1.27) | -0.54 (-2.92,1.83) | 0.80 (-1.73,3.32) |
| Age 18yr (bpm) | 61.75 (61.23,62.27) | -0.22 (-1.37,0.93) | | 2.84 (-0.82,6.51) | 0.17 (-2.74,3.08) | -1.54 (-5.03,1.94) | 3.39 (-0.28,7.06) |

bpm, beats per minute; bpm/yr, beats per minute per year; CI, confidence interval; mmHg, millimetres of mercury; mmHg/yr, millimetres of mercury per year. For details of the specific subgroups contributing to these larger groups see Supplementary Table 1. The “other” haplogroup includes (listed in order of frequency): T, Q, H, L, C, N and O.

**Table 18 Mean trajectories of glucose estimated from multilevel models, by haplogroup**

|  | Mean trajectory (95% CI) in haplogroup R (reference) | Mean difference in trajectory (95% CI) comparing haplogroup I with haplogroup R | Mean difference in trajectory (95% CI) comparing haplogroup E with haplogroup R | Mean difference in trajectory (95% CI) comparing haplogroup J with haplogroup R | Mean difference in trajectory (95% CI) comparing haplogroup G with haplogroup R | Mean difference in trajectory (95% CI) comparing all other haplogroups combined with haplogroup R |
| --- | --- | --- | --- | --- | --- | --- |
|  |  |  |  |  |  |  |
| Glucose |  |  |  |  |  |  |
| Age 7yr (mmol/l) | 4.34 (4.32,4.36) | -0.01 (-0.06,0.03) | -0.10 (-0.21,0.02) | -0.05 (-0.16,0.06) | 0.04 (-0.09,0.17) | -0.03 (-0.18,0.12) |
| Change 7-15yr (mmol/l/yr) | 0.12 (0.12,0.13) | 0.003 (-0.01,0.01) | 0.004 (-0.02,0.03) | 0.01 (-0.01,0.03) | -0.01 (-0.03,0.02) | 0.003 (-0.02,0.03) |
| Change 15-18yr (mmol/l/yr) | -0.07 (-0.09,-0.06) | 0.01 (-0.02,0.03) | 0.02 (-0.05,0.08) | -0.03 (-0.09,0.04) | 0.01 (-0.06,0.09) | 0.001 (-0.08,0.08) |
| Age 18yr (mmol/l) | 5.12 (5.09,5.14) | 0.03 (-0.03,0.08) | -0.01 (-0.15,0.12) | -0.05 (-0.19,0.10) | 0.005 (-0.14,0.15) | -0.003 (-0.17,0.17) |

CI, confidence interval; mmol/l, millimole per litre; mmol/l/year, millimoles per litre per year. For details of the specific subgroups contributing to these larger groups see Supplementary Table 1. The “other” haplogroup includes (listed in order of frequency): T, Q, H, L, C, N and O.

Table 19 Mean trajectories of triglycerides estimated from multilevel models, by haplogroup

|  | **Mean trajectory (95% CI) in haplogroup R (mmol/l or mmol/l/yr) (reference)** ^a^ | **Mean difference in trajectory (95% CI) comparing haplogroup I with haplogroup R (% or %/yr)** ^b^ | **Mean difference in trajectory (95% CI) comparing haplogroup E with haplogroup R(% or %/yr)** ^b^ | **Mean difference in trajectory (95% CI) comparing haplogroup J with haplogroup R(% or %/yr)** ^b^ | **Mean difference in trajectory (95% CI) comparing haplogroup G with haplogroup R(% or %/yr)** ^b^ | **Mean difference in trajectory (95% CI) comparing other haplogroups with haplogroup R(% or %/yr)** ^b^ |
| --- | --- | --- | --- | --- | --- | --- |
|  |  |  |  |  |  |  |
| **Log triglycerides** |  |  |  |  |  |  |
| Birth (mmol/l or %) | -0.67 (-0.70,-0.65) | -3.43 (-8.49,1.63) | -8.48 (-20.23,3.26) | -9.62 (-22.11,2.88) | 2.7 (-13.57,18.94) | 15.94 (-2.23,34.10) |
| Change 0-9yr (mmol/l/yr or %/yr) | 0.08 (0.07,0.08) | 0.54 (-0.21,1.30) | 0.65 (-1.14,2.45) | 0.52 (-1.42,2.47) | 0.2 (-2.03,2.41) | -2.90 (-5.12,-0.67) |
| Change 9-18yr (mmol/l/yr or %/yr) | -0.04 (-0.04,-0.04) | -0.24 (-0.87,0.40) | -0.54 (-2.01,0.92) | -0.01 (-1.56,1.55) | 0.2 (-1.57,1.91) | -0.01 (-1.56,1.55) |
| Age 18yr (mmol/l or %) | -0.33 (-0.35,-0.31) | -0.75 (-5.14,3.63) | -7.62 (-17.17,1.92) | -30.67 (-50.65,-10.69) | 6.0 (-6.66,18.65) | -3.06 (-15.87,9.74) |
|  |  |  |  |  |  |  |

CI, confidence interval; mmol/l, millimole per litre; mmol/l/year, millimoles per litre per year; %/yr, percentage per year

^a^ Triglycerides were transformed using the natural log. All predicted mean values (mmol/l) and rates of change per year (mmol/l/yr) are on the log scale

^b^ *The difference between haplogroups is back transformed from the log scale for ease of interpretation and is interpreted as the percentage difference in the mean level comparing each category with haplogroup R or percentage difference in change per year (%/yr) comparing each category with haplogroup R. For details of the specific subgroups contributing to these larger groups see Supplementary Table 1. The “other” haplogroup includes (listed in order of frequency): T, Q, H, L, C, N and O.

Table 20 Mean trajectories of cholesterol estimated from multilevel models, by haplogroup

|  | **Mean trajectory (95% CI) in haplogroup R (reference)** | **Mean difference in trajectory (95% CI) comparing haplogroup I with haplogroup R** | **Mean difference in trajectory (95% CI) comparing haplogroup E with haplogroup R** | **Mean difference in trajectory (95% CI) comparing haplogroup J with haplogroup R** | **Mean difference in trajectory (95% CI) comparing haplogroup G with haplogroup R** | **Mean difference in trajectory (95% CI) comparing all other haplogroups combined with haplogroup R** |
| --- | --- | --- | --- | --- | --- | --- |
|  |  |  |  |  |  |  |
| **HDL-c** |  |  |  |  |  |  |
| Birth (mmol/l) | 0.51 (0.50,0.52) | -0.02 (-0.04,0.01) | -0.06 (-0.12,0.01) | 0.01 (-0.06,0.08) | -0.04 (-0.12,0.04) | 0.01 (-0.07,0.09) |
| Change 0-7yr (mmol/l/yr) | 0.15 (0.14,0.15) | 0.001 (-0.004,0.01) | 0.01 (-0.001,0.02) | 0.005 (-0.01,0.02) | 0.01 (-0.01,0.02) | 0.003 (-0.01,0.02) |
| Change 7-18yr (mmol/l/yr) | -0.04 (-0.04,-0.03) | -0.001 (-0.004,0.002) | -0.003 (-0.01,0.004) | -0.004 (-0.01,0.003) | -0.004 (-0.01,0.004) | -0.004 (-0.01,0.003) |
| Age 18yr (mmol/l) | 1.15 (1.14,1.16) | -0.02 (-0.04,0.01) | -0.004 (-0.07,0.06) | -0.02 (-0.17,0.13) | -0.02 (-0.10,0.06) | 0.07 (-0.01,0.16) |
| **Non-HDL-c** |  |  |  |  |  |  |
| Birth (mmol/l) | 1.23 (1.20,1.26) | -0.03 (-0.09,0.03) | -0.15 (-0.30,-0.01) | 0.02 (-0.13,0.18) | -0.05 (-0.23,0.13) | 0.03 (-0.17,0.22) |
| Change 0-9yr (mmol/l/yr) | 0.19 (0.19,0.20) | 0.001 (-0.01,0.01) | 0.01 (-0.01,0.03) | -0.01 (-0.03,0.02) | -0.0005 (-0.03,0.03) | -0.02 (-0.05,0.01) |
| Change 9-18yr (mmol/l/yr) | -0.07 (-0.08,-0.07) | 0.004 (-0.003,0.01) | -0.01 (-0.02,0.01) | -0.01 (-0.03,0.01) | 0.0002 (-0.02,0.02) | -0.01 (-0.03,0.01) |
| Age 18yr (mmol/l) | 2.30 (2.27,2.33) | 0.02 (-0.05,0.09) | -0.14 (-0.29,0.01) | -0.26 (-0.62,0.09) | -0.05 (-0.23,0.13) | -0.13 (-0.33,0.08) |
|  |  |  |  |  |  |  |

CI, confidence interval; mmol/l, millimole per litre; mmol/l, millimole per litre per year. For details of the specific subgroups contributing to these larger groups see Supplementary Table 1. The “other” haplogroup includes (listed in order of frequency): T, Q, H, L, C, N and O.

Table 21 Mean trajectories of CRP estimated from multilevel models, by haplogroup

|  | **Mean trajectory (95% CI) in log CRP haplogroup R (reference) (mgl/l)** ^a^ | **Mean difference in trajectory (95% CI) comparing haplogroup I with haplogroup R (% difference in mg/l)** ^b^ | **Mean difference in trajectory (95% CI) comparing haplogroup E with haplogroup R (% difference in mg/l)** ^b^ | **Mean difference in trajectory (95% CI) comparing haplogroup J with haplogroup R (% difference in mg/l)** ^b^ | **Mean difference in trajectory (95% CI) comparing haplogroup G with haplogroup R (% difference in mgl/l)** ^b^ | **Mean difference in trajectory (95% CI) comparing all other haplogroups combined with haplogroup R (% difference in mgl/l)** ^b^ |
| --- | --- | --- | --- | --- | --- | --- |
|  |  |  |  |  |  |  |
| **Log CRP** |  |  |  |  |  |  |
| Age 9 (mg/l or %) | -1.51 (-1.57,-1.46) | 10.20 (-3.01,23.40) | -2.92 (-28.49,22.66) | 2.58 (-26.80,31.95) | 20.3 (-19.48,60.09) | 49.15 (-8.88,107.18) |
| Age 15 (mg/l or %) | -0.87 (-0.92,-0.83) | -4.48 (-14.56,5.60) | -5.28 (-27.68,17.11) | 24.81 (-7.64,57.27) | -19.4 (-42.41,3.58) | -9.94 (-39.87,19.99) |
| Age 18 (mgmol/l or %) | -0.49 (-0.55,-0.44) | -7.07 (-22.77,8.62) | -8.32 (-43.00,26.37) | 42.56 (-16.75,101.88) | -29.2 (-61.53,3.11) | -15.42 (-60.39,29.55) |
|  |  |  |  |  |  |  |

CI, confidence interval; CRP, c-reactive protein; mg/l, milligram per litre.

^a^ CRP was transformed using the natural log. All predicted mean values for haplogroup R are on the log scale.

^b^ The difference between haplogroups is back transformed from the log scale for ease of interpretation and is interpreted as the percentage difference in the mean level comparing each category with haplogroup R. For details of the specific subgroups contributing to these larger groups see Supplementary Table 1. The “other” haplogroup includes (listed in order of frequency): T, Q, H, L, C, N and O.


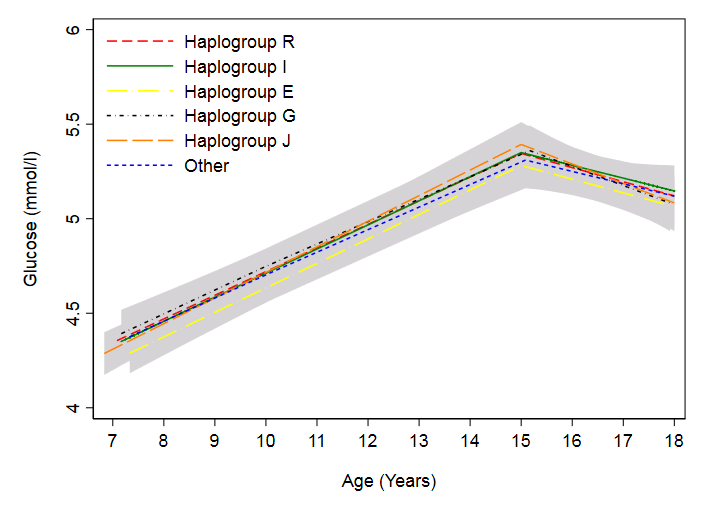


N=3,020

Figure 1 Mean predicted trajectories of glucose from 7 to 18 years, excluding participants who reported eating before either the 15-or 18-year clinic by haplogroup

For details of the specific subgroups contributing to these larger groups see Supplementary Table 1. The “other” haplogroup includes (listed in order of frequency): T, Q, H, L, C, N and O. Confidence intervals for all haplogroups are displayed in grey but are entirely over-lapping as the difference between the trajectories of the haplogroups spans the null value across the entire age range.


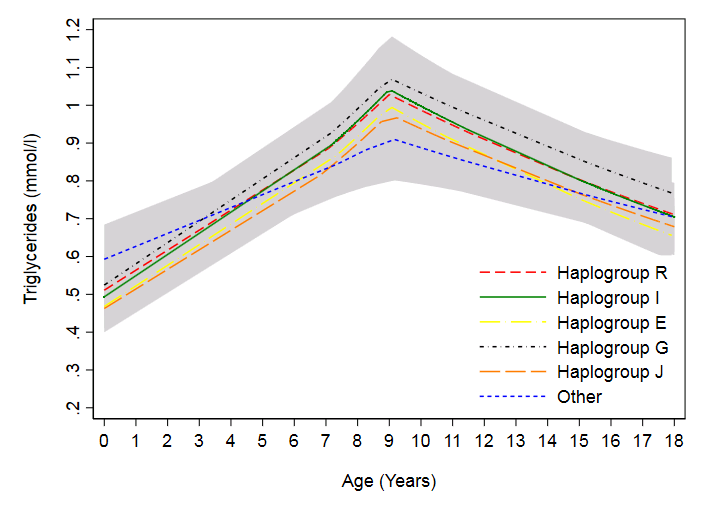
**
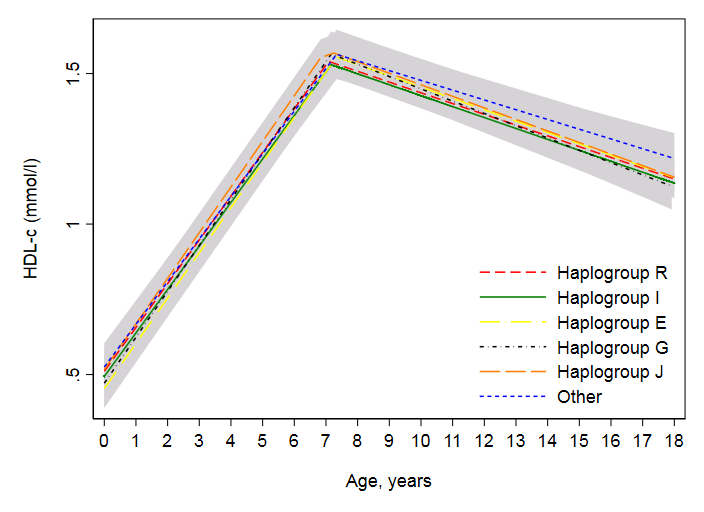
**
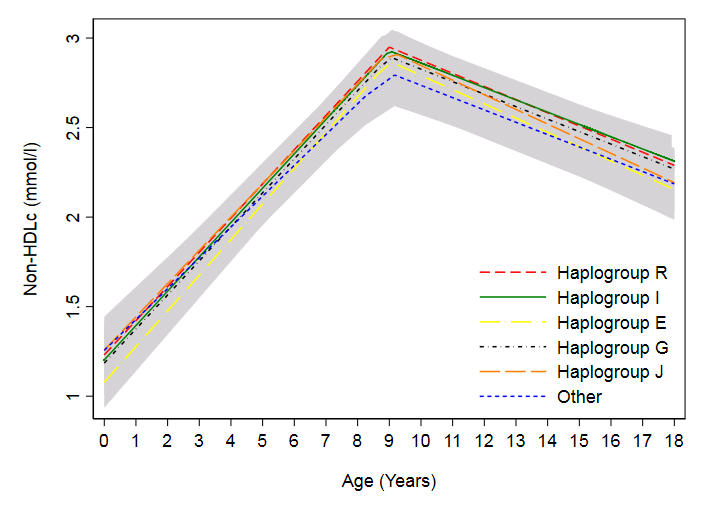


**A B C**

**N=4,172 N=4,172 N=4,172**

Figure 2 Mean predicted trajectories of lipids from birth to 18 years excluding participants who reported eating before either the 15 or 18-year clinic by haplogroup

Mean predicted trajectories of (A) triglyceride, (B) HDL-c (C) and non-HDL-c by haplogroup

HDL-c, high density lipoprotein cholesterol; non-HDL-c, non-high density lipoprotein cholesterol. For details of the specific subgroups contributing to these larger groups see Supplementary Table 1. The “other” haplogroup includes (listed in order of frequency): T, Q, H, L, C, N and O. Confidence intervals for all haplogroups are displayed in grey but are entirely over-lapping as the difference between the trajectories of the haplogroups spans the null value across the entire age range.

**
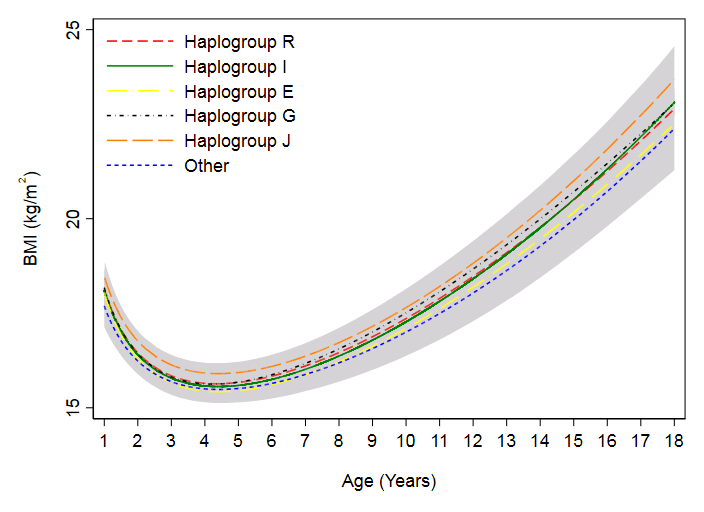
**

**N=3,455**

Figure 3 Mean predicted trajectory of BMI from 1-18 years among participants with 6 or more measures by haplogroup

For details of the specific subgroups contributing to these larger groups see Supplementary Table 1. The “other” haplogroup includes (listed in order of frequency): T, Q, H, L, C, N and O. Confidence intervals for all haplogroups are displayed in grey but are entirely over-lapping as the difference between the trajectories of the haplogroups spans the null value across the entire age range.

**References**

1. Price AL, Patterson NJ, Plenge RM, Weinblatt ME, Shadick NA, Reich D. Principal components analysis corrects for stratification in genome-wide association studies. Nature genetics. 2006;38(8):904-9.

2. Purcell S, Neale B, Todd-Brown K, Thomas L, Ferreira MA, Bender D, et al. PLINK: a tool set for whole-genome association and population-based linkage analyses. The American Journal of Human Genetics. 2007;81(3):559-75.

3. Howe LD, Tilling K, Lawlor DA. Accuracy of height and weight data from child health records. Archives of disease in childhood. 2009;94(12):950-4.

4. Howe LD, Parmar PG, Paternoster L, Warrington NM, Kemp JP, Briollais L, et al. Genetic Influences on Trajectories of Systolic Blood Pressure Across Childhood and Adolescence. Circulation: Cardiovascular Genetics. 2013;6(6):608-14.

5. Morris T, Northstone K, Howe L. Examining the association between early life social adversity and BMI changes in childhood: a life course trajectory analysis. Pediatric obesity. 2015.
